# Does the Method of Weight Loss Effect Long-Term Changes in Weight, Body Composition or Chronic Disease Risk Factors in Overweight or Obese Adults? A Systematic Review

**DOI:** 10.1371/journal.pone.0109849

**Published:** 2014-10-15

**Authors:** Richard A. Washburn, Amanda N. Szabo, Kate Lambourne, Erik A. Willis, Lauren T. Ptomey, Jeffery J. Honas, Stephen D. Herrmann, Joseph E. Donnelly

**Affiliations:** 1 Cardiovascular Research Institute, Department of Internal Medicine, The University of Kansas Medical Center, Kansas City, Kansas, United States of America; 2 Sanford Research, Department of Children's Health Research Center, Sioux Falls, South Dakota, United States of America; Pennington Biomed Research Center, United States of America

## Abstract

**Background:**

Differences in biological changes from weight loss by energy restriction and/or exercise may be associated with differences in long-term weight loss/regain.

**Objective:**

To assess the effect of weight loss method on long-term changes in weight, body composition and chronic disease risk factors.

**Data Sources:**

PubMed and Embase were searched (January 1990-October 2013) for studies with data on the effect of energy restriction, exercise (aerobic and resistance) on long-term weight loss. Twenty articles were included in this review.

**Study Eligibility Criteria:**

Primary source, peer reviewed randomized trials published in English with an active weight loss period of >6 months, or active weight loss with a follow-up period of any duration, conducted in overweight or obese adults were included.

**Study Appraisal and Synthesis Methods:**

Considerable heterogeneity across trials existed for important study parameters, therefore a meta-analysis was considered inappropriate. Results were synthesized and grouped by comparisons (e.g. diet vs. aerobic exercise, diet vs. diet + aerobic exercise etc.) and study design (long-term or weight loss/follow-up).

**Results:**

Forty percent of trials reported significantly greater long-term weight loss with diet compared with aerobic exercise, while results for differences in weight regain were inconclusive. Diet+aerobic exercise resulted in significantly greater weight loss than diet alone in 50% of trials. However, weight regain (∼55% of loss) was similar in diet and diet+aerobic exercise groups. Fat-free mass tended to be preserved when interventions included exercise.

## Introduction

An energy restricted diet, aerobic exercise, and diet combined with aerobic exercise, have shown success in producing clinically significant (≥5%) [1] reductions in body weight [2]–[7]. However, the combined prevalence of overweight and obesity among US adults continues at approximately 69% [8], due in part to the inability of individuals who lose weight to maintain weight loss long term [9]–[14].

Nearly 25 years ago Pavlou et al [15] observed that aerobic exercise combined with an energy restricted diet during weight loss was essential for weight loss maintenance. It has been hypothesized that the type and magnitude of biological changes predisposing to weight regain may differ between weight loss induced by diet compared with aerobic exercise [16] which may explain the observation of Pavlou. Several excellent reviews have described the compensatory changes in biological systems involved in energy utilization/storage and appetite regulation induced by an energy restricted diet [17]–[21]. These changes act in concert to predispose individuals to regain lost weight. For example, weight loss by diet results in decreased resting and non-resting energy expenditure [22]–[24], fat oxidation [25], thyroid hormones [26]–[28] and increased cortisol [29], [30]; changes which all are associated with increased energy storage. Energy restriction also results in increased gastric inhibitory peptide and ghrelin [31], decreased leptin [32], peptide YY [33], [34], amylin, and insulin [35] and alterations in the sympathetic nervous system [36]–[38] that all promote increased energy intake. Changes in several of these hormones [32] as well as resting energy expenditure [39] may not be transient, but may persist for up to one year post diet induced weight loss. Given the complexity of the control of energy balance, which involves psychological, physiological and environmental factors, weight loss induced changes in any one of physiological parameters is unlikely to be individually predictive of weight regain [19].

In contrast to weight loss by an energy restricted diet alone, weight loss via aerobic exercise or a combination of diet and aerobic exercise results in beneficial changes on both sides of the energy balance equation which may be associated with better maintenance of lost weight. For example, weight loss by an energy restricted diet is associated with decreased resting metabolic rate (RMR) [40], [41] which may [24], [42], [43] or may not [22], [44], [45] be greater than expected based on the loss of fat and fat-free mass. In contrast, weight loss achieved by aerobic exercise is composed predominantly of fat mass, while fat-free mass is preserved [6], [7], [46], and RMR is generally unchanged [47] or slightly increased [48]. In addition, reductions in RMR/kg weight loss may be greater for weight loss induced by energy restriction compared with weight loss by exercise [49] and potential metabolic adaptation in RMR may be eliminated when exercise is combined with energy restriction [43], [50]. Weight loss by energy restriction has also been shown to result in significant decreases in both total daily energy expenditure and physical activity energy expenditure [51], [52] which are eliminated when energy restriction is combined with aerobic exercise [11], [43].

Aerobic exercise may also improve the coupling between energy intake and energy expenditure [16], [53]–[57] which may be important in maintaining energy balance in a weight reduced state. In contrast, physical inactivity disrupts the homeostatic mechanisms associated with appetite control which could contribute to positive energy balance and weight gain [53], [58]–[61]. While the mechanisms for this exercise effect are unclear, alterations in appetite regulatory hormones [62] [63] and reduced neuronal response in brain regions known to be associated with food intake regulation in a direction expected to lead to reduced EI [64] have been hypothesized.

Several earlier reviews/meta-analyses have provided evidence to support the hypothesis of better long-term weight loss for interventions using energy restriction plus exercise compared with energy restriction alone [65]–[67] while others have not [68]–[70]. The discrepancy in results among reviews is likely a function of differences in inclusion criteria in terms of study designs (observational vs. randomized trials), length of weight loss/follow-up periods, and participant characteristics including age, sex and baseline BMI. The objective of this systematic review was to update and expand upon previous reviews by identifying and evaluating randomized trials that have compared the effect of energy restriction, exercise (aerobic and resistance) and there various combinations, on long-term weight loss, changes in body composition and chronic disease risk factors.

## Methods

This systematic review was performed and reported in accordance with the Preferred Reporting Items for Systematic Reviews and Meta-Analysis (PRISMA) guidelines [71], [72].

### Objectives

The objective of this systematic review was to address the question:

Does the method of weight loss, i.e., energy restriction, exercise (aerobic or resistance), and there various combinations effect long-term changes in weight, body composition or chronic disease risk factors in overweight or obese adults?

### Eligibility criteria

Only primary source articles published in English in peer-reviewed journals utilizing a randomized design were included. Specific eligibility included: *Participants*: Overweight and obese (BMI≥25 kg/m^2^) adults (age 18–65 yrs). Studies of participants with elevated chronic disease risk factors and/or type 2 diabetes (DM2) were included. *Types of studies*: Our original intent was to evaluate trials that included at least a 6 month active weight loss intervention followed by at least 12 months of active or passive weight maintenance. We were able to identify only 3 randomized trials that satisfied these criteria. Therefore, we expanded our criteria to include trials with an active weight loss and weight maintenance phase of any duration, as well as trials with an active weight loss intervention longer than 6 months. *Comparisons*: Weight loss by energy restriction alone, exercise alone (aerobic or resistance), and there various combinations with the comparisons in the same randomized trial. *Outcomes*: Body weight was required for inclusion. Other outcomes included, if available, were body composition (fat mass, fat-free mass, waist circumference) and chronic disease risk factors including total, HDL and LDL cholesterol, triglycerides, insulin, glucose, HbA_1c_, and blood pressure.

### Information Sources

Studies were identified by searching electronic data bases, related article reference lists, and consulting with experts in the field. The search was applied to PubMed (1990 – present) and adapted for EMBASE (1990—present). The last search was conducted on October 31^st^ 2013. The search was developed as a collaborative effort of the research team in consultation with a Kansas University reference librarian and conducted by a co-author (AS). No attempts were made to contact study investigators or sponsors to acquire any information missing from the published article.

### Search Strategy

Studies were identified by searching electronic data bases, related article reference lists, and consulting with experts in the field. The search was applied to PubMed (1990 – present) and adapted for EMBASE (1990—present). The last search was conducted on October 31^st^, 2013. The search was developed as a collaborative effort of the research team in consultation with a Kansas University reference librarian and conducted by a co-author (AS). No attempts were made to contact study investigators or sponsors to acquire any information missing from the published article.

### Search Strategy

We used the following search terms for Pub Med to identify potential articles with abstracts for review: Physical Activity[Title/Abstract] OR Exercise[Title/Abstract] OR diet[Title/Abstract] OR calorie restriction[Title/Abstract] OR low calorie diet[Title/Abstract]) AND (weight loss[Title/Abstract] OR weight maintenance[Title/Abstract] OR weight regain[Title/Abstract]) NOT spinal[All Fields] NOT paraplegia[Title/Abstract] NOT stroke[Title/Abstract] NOT athletes[Title/Abstract] NOT Alzheimer's[Title/Abstract] NOT fibromyalgia[Title/Abstract] NOT wheelchair[Title/Abstract] NOT "surgical procedures, operative"[All Fields] NOT "general surgery"[MeSH Terms] NOT cancer[Title/Abstract] NOT dialysis[Title/Abstract] NOT pregnant[Title/Abstract] NOT injury[Title/Abstract] NOT HIV[Title/Abstract] NOT Parkinson[Title/Abstract] NOT depression[Title/Abstract] NOT Cancer[Title/Abstract] NOT pregnant[Title/Abstract] AND (("1990/01/01"[PDAT]: "3000/12/31"[PDAT]) AND "humans"[MeSH Terms] AND English[lang] AND ("adult"[MeSH Terms:noexp] OR "middle aged"[MeSH Terms])) AND (Clinical Trial[ptyp] OR Randomized Controlled Trial[ptyp]).

The search was then modified slightly for EMBASE: 'physical activity'/exp OR 'physical activity' OR 'exercise'/exp OR 'exercise' OR 'diet'/exp OR diet OR 'calorie restriction'/exp OR 'calorie restriction' OR 'low calorie' AND ('weight loss'/exp OR 'weight loss' OR 'weight maintenance' OR 'weight regain') NOT (spinal OR paraplegia OR depression OR stroke OR athletes OR fibromyalgia OR wheelchair OR pregnantOR lactation OR alzheimer OR parkinson OR 'cancer'/exp OR 'surgery'/exp OR 'general surgery') AND [adult]/lim AND [humans]/lim AND [embase]/lim AND [1990–2014]/py AND ([controlled clinical trial]/lim OR [randomized controlled trial]/lim) AND [english]/lim AND [2000–2014]/py. Word truncation and the use of wildcards allowed for variations in spelling and word endings.

### Study Selection

Retrieved abstracts were independently assessed for inclusion in the review by 2 investigators and coded as “yes”, “no” or “maybe.” All investigators who participated in eligibility assessments were trained regarding study inclusion/exclusion criteria and completed practice eligibility assessments on 50 test abstracts prior to actual coding. Eligibility assessments on the practice abstracts were reviewed by the primary author (JED) and any coding problems were resolved. Disagreements regarding eligibility for inclusion were resolved via development of consensus among all co-authors. Full text articles for abstracts coded as “yes” or “maybe” were retrieved and reviewed independently by 2 co-authors prior to inclusion in the review. An excel spread sheet was developed and used to track eligibility status.

### Data collection

Extracted data was entered into the University of Kansas secure, REDCap (Research Electronic Data Capture, Version 4.14.5) data base [73]. A REDCap data extraction form was developed, pilot tested on a sample of 10 studies and revised accordingly. Relevant data were extracted from each manuscript by one author and verified by a second author. Disagreements were resolved by discussion. Data extracted from each article included basic study information including design (long-term or weight loss with follow-up), sample size, groups compared, diet intervention, exercise intervention, compliance with the diet and exercise interventions, participant characteristics (age, sex, BMI, minority and health status), and results.

### Risk of bias in individual studies

Risk of bias for randomized trials was independently evaluated by two authors using the Cochrane risk of bias tool [74]. Risk of bias was assessed in the following domains: selection bias, performance bias, detection bias, attrition bias, reporting bias, and other bias. A third reviewer resolved any discrepancies in bias coding. Studies were not excluded on the basis of risk of bias.

### Synthesis of results

There was considerable heterogeneity across studies for several important parameters including: 1) participant characteristics (age, sex, BMI, health status), 2) diet (low calorie, very low calorie) and exercise prescriptions (mode, frequency, intensity, duration), 3) comparison groups (diet, aerobic exercise, resistance exercise and all possible combinations), 4) duration of the weight loss and/or follow periods, and type of intervention during the follow-up period. Given this heterogeneity, a meta-analysis was considered inappropriate. Results based on the extracted data were instead synthesized and presented grouped by comparisons (e.g. diet vs. aerobic exercise, diet vs. diet plus aerobic exercise etc.) and study design (long-term or weight loss with follow-up).

## Results

**Figure 1:**
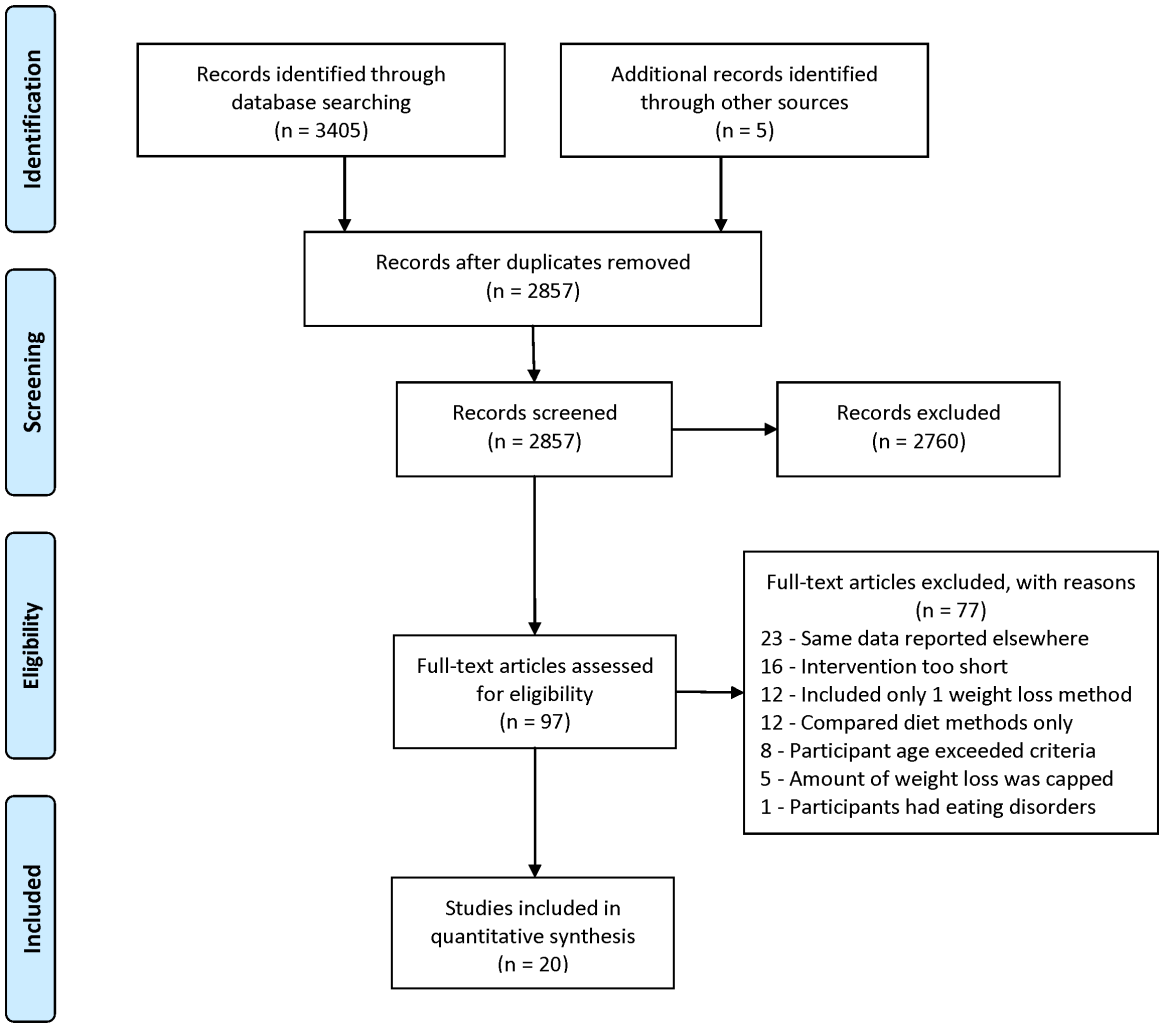
Flow diagram for identification, screening, assessing eligibility, and inclusion in systematic review.

The initial database search plus hand searching identified 2,857 unique records of which 2,760 were excluded based on review of title and abstract. Full-text articles for the remaining 97 citations were reviewed. Seventy seven articles did not satisfy our inclusion criteria and were excluded, thus 20 articles were included in the review (Figure 1). Six trials (30%) reported on long-term weight loss while 14 trials (70%) reported on weight loss with follow-up assessments. Study and participant characteristics are presented in Tables 1 and 2.

**Table 1:** Study Characteristics

Study	Intervention groups	Sample size/sex/health status/minority %	Age (yrs.)	BMI (kg/m^2^)	Length weight loss	Length follow-up	Outcomes
							
**Long-term trials**							
Anderssen et al.(75)	D, AE, D+AE, C	D = 52	NR by group.	D = 29.5 (0.5) a	12 mos.	NA	WT, WC, BP, TC, HDL-C, LDL-C, TG
		AE = 49	Mean age ∼40 yrs.	AE = 28.6 (0.5)			
		D+AE = 65		D+AE = 28.6 (0.4)			
		C = 43		C = 28.3 (0.5)			
		9.6% women					
		High BMI, DBP, TC, TG, low HDL-C					
		Minority-NR					
Dengel et al.(81)	D, D+AE, C	D = 85	D = 61.0 (1.7-SE)	D = 30.1 (0.5-SE)	10 mos.	NA	WT, BC (under water weight)
		D+AE = 37	D+AE = 57.1(1.4-SE)	D+AE = 30.1(0.6-SE)			
		C = 16	C = 63.6 (1.9-SE)	C = 29.5 (0.8-SE)			
		100% men					
		Healthy					
		Minority-0%					
Foster-Schubert et al. (76)	D, AE, D+AE, C	D = 118	D = 58.1 (6.0)	D = 31.1(3.9)	12 mos.	NA	WT, WC, BC (DXA)
		AE = 117	AE = 58.1 (5.0)	AE = 30.7(3.7)			
		D+AE = 117	D+AE = 58.0 (4.5)	D+AE = 31.0 (4.3)			
		C = 87	C = 57.4 (4.4)	C = 28.6 (2.8)			
		100% women					
		Healthy					
		Minority-15%					
Pritchard et al. (78)	D, AE, C	D = 18	D = 42.3(4.5)	D = 29.0(2.8)	12 mos.	NA	WT, BC (DXA)
		AE = 21	AE = 44.9(6.5)	AE = 29.2(2.8)			
		C = 19	C = 42.3(4.5)	C = 28.6(2.8)			
							
		100% men					
		Healthy					
		Minority-NR					
Racette et al. (5)	D, AE, C	D = 21	D = 55.6(0.8-SE)	D = 27.2 (0.6-SE)	12 mos.	NA	WT, BC (DXA/MRI)
		AE = 24	AE = 58.8(0.6-SE)	AE = 27.2(0.4-SE)			
		C = 13	C = 56.0(0.9-SE)	C = 27.9(0.4-SE)			
		62% women					
		Healthy					
		Minority-10.2%					
Wood et al. (82)	D, D+AE, C	D = 67	NR by group	NR by group	12 mos.	NA	WT, TC, HDL-C, LDL-C, TG, BP, BC (underwater weight)
		D+AE = 90	Men = 40.3 (6.3)	Men = 30.7 (2.2)			
		C = 87	Women = 39.1 (6.4)	Women = 27.9 (2.2)			
		49% women					
		Healthy					
		Minority-NR					
**Follow-up trials**							
Andreou et al. (83)	D, D+AE	D = 95	D = 33.0(10.1)	D = 31.4(5.9)	18 wks.	18 wks.	WT, WC, BC, (BIA)
		D+AE = 111	D+AE = 35 (11.2)	D+AE = 29.1(4.8)			
							
		100% women					
		Healthy					
		Minority-All from Cyprus					
Hunter et al. (84)	D, D+AE, D+RE	D = 30	D = 34.8 (5.6)	D = 29.3(1.1)^3^	154 days	12 mos.	WT, BC (DXA and MRI)
		D+AE^1^ = 18	D+AE^1^ = 34.1(7.2)	D+AE^1^ = 23.5(1.0)			
		D+AE^2^ = 14	D+AE^2^ = 34.1 (5.3)	D+AE^2^ = 24.1(1.2)			
		D+RE^1^ = 21	D+RE^1^ = 34.7 (8.4)	D+RE^1^ = 23.9 (1.0)			
		D+RE^2^ = 14	D+RE^2^ = 36.2 (6.6)	D+RE^2^ = 24.0 (1.2)			
							
		100% women					
		Healthy					
		Minority-54%					
Melanson et al. 2004 (91)	AE, D+AE	AE = 47	NR by group	AE = 30.6(0.5-SE)	12 wks.	12 wks.	WT, WC. BC(air displacement) TC, HDL-C,LDL-C, TG
		D+AE = 42	Mean age = 42.6(6.0)	D+AE+31.8(0.7-SE)			
							
		88% women					
		Healthy					
		Minority-NR					
Melanson et al 2006 (92)	AE, D+AE(cereal),	AE = 43	AE = 42.6(1.4-SE)	AE = 31.0(2.4-SE)	12 wks.	12 wks.	WT
	D+AE(no cereal)	D+AE(cereal) = 45	D+AE(cereal) = 42.5 (0.9-SE)	D+AE(cereal) = 31.1(2.5-SE)			
		D+AE(no cereal) = 46	D+AE(no cereal) = 41.7(1.2-SE)	D+AE(no cereal) = 30.5(2.4-SE)			
							
		79% women					
		Healthy					
		Minority-25.9%					
Molenar et al. (85)	D, D+AE, C	D = 67	D = 43 (9)	D = 31.3(2.0)	6 mos.	6 mos.	WT, WC
		D+AE = 67	D+AE = 43(10)	D+AE = 30.8(1.9)			
		C = 70	C = 41(11)	C = 30.2(1.9)			
							
		40% women					
		Healthy					
		Minority-NR					
Neumark-Sztainer et al. (86)	D, D+AE	D = 21	NR by group	NR by group	3 mos.	5 mos.	WT, WC, BC(skinfolds)
		D+AE = 21	Age range = 25-50	Mean BMI = 30.3 (SD-NR)			
							
		100% women					
		Healthy					
		70% Asian/North African					
Skender et al. (77)	D, AE, D+AE	D = 42	NR by group	NR	12 mos.	12 mos.	WT
		AE = 43	Age range (25-45)				
		D+AE = 42					
							
		47% women					
		Healthy					
		Minority-NR					
Snel et al. (87)	D, D+AE	D = 13	D = 59.0 (2.0-SE)	D = 37.9(1.4-SE)	4 mos.	14 mos.	WT, WC, BC (BIA), TC, HDL-C, LDL-C, TG, HbA_1C_, INSULIN, GLUCOSE,
		D+AE = 14	D+AE = 56 (2.0-SE)	D+AE = 36.4(1.1-SE)			
							
		47% women					
		Insulin dependent type 2 diabetics. Off medicine during weight loss.					
		Minority-0%					
							
Svendsen et al. (93)	D, D+AE+RE, C	D = 51	NR by group	NR by group	12 wks.	6 mos.	WT,BC (DXA) TC, HDL-C, LDL-C, TG
		D+AE+RT = 49	Sample mean = 53.8 (2.5)	Sample mean = 29.7(3.1)			
		C = 21					
							
		100% women					
		Healthy					
		Minority-NR					
Van Aggel-Leijssen et al. (88)	D,D+AE	D = 17	D = 38.6(6.5)	D = 32.0 (2.1)	12 wks.	40 wks.	WT, BC (underwater weight)
		D+AE = 20	D+AE = 39.3(7.7)	D+AE = 32.6 (2.5)			
							
		100% men					
		Healthy					
		Minority-NR					
Volpe et al.(79)	D, AE, D+AE	Women	Women	Women	6 mos.	6 mos.	WT, WC, BC (underwater weight), BP, TC, HDL-C, LDL-C, TG
		D = 15	D = 44.0(6.5)	D = 30.8(2.8)			
		AE = 17	AE = 42.4(7.3)	AE = 30.5(3.0)			
		D+AE = 14	D+AE = 45.1(4.8)	D+AE = 30.4(2.5)			
							
		Men	Men	Men			
		D = 13	D = 43.9(6.5	D = 31.1(3.3)			
		AE = 17	AE = 44.6(8.1)	AE = 30.5(3.6)			
		D+AE = 14	D+AE = 44.9(9.8)	D+AE = 30.5(3.2)			
		Healthy					
		Minority-NR					
Wadden et al. (89)	D, D+AE, D+RE, D+AE+RE	D = 29	D = 41.0 (8.8)	D = 36.4 (5.5)	48 wks.	52 wks.	WT, BC (underwater weight)
		D+AE = 31	D+AE = 40.8 (7.9)	D+AE = 37.3(5.1)			
		D+RE = 31	D+RE = 40.0 (9.1)	D+RE = 36.5 (6)			
		D+AE+RE = 29	D+AE+RE = 42.8 (8.0)	D+AE+RE = 35.3(4.4)			
							
		100% women					
		Healthy					
		Minority-30%					
							
Wang et al. (90)	D, D+AE(low intensity), D+AE(high intensity)	D = 15	NR by group	BMI NR	20 wks.	52 wks.	WT
		D+AE(low intensity) = 13	Sample mean = 58.6 (2.5)	%fat by group			
		D+AE(high intensity) = 13		D = 43(4)			
				D+AE(low intensity) = 42.3(3.8)			
		100% women		D+AE(high intensity) = 43.1(3.4)			
		Healthy					
		Minority-34%					
Wing et al. (80)	D, AE, D+AE, C	D = 37	D = 45.0 (4.7)	D = 36.1(4.1)	6 mos.	18 mos.	WT, BP, TC, HDL-C, LDL-C, TG, HbA1C, INSULIN, GLUCOSE
		AE = 37	AE = 46.4(4.5)	AE = 36.0(3.7)			
		D+AE = 40	D+AE = 46.3(3.8)	D+AE = 35.7(4.1)			
		C = 40	C = 45.3(4.9)	C = 36.0(5.4)			
							
		79% women					
		Healthy with 1 or 2 biological parents with type 2 diabetes					
		Minority-NR					

a Values are mean (SD) unless otherwise stated.

1 Adhered to exercise protocol.

2 Did not adhere to exercise protocol.

3 Post weight loss.

Abbreviations: AE  =  aerobic exercise, D  =  diet, D+AE  =  diet plus aerobic exercise, RE  =  resistance exercise, D+RE  =  diet plus resistance exercise, D+AE+RE  =  diet plus aerobic plus resistance exercise, C  =  control, BC  =  body composition, BMI  =  body mass index, BP  =  blood pressure, DBP  =  diastolic blood pressure, DXA  =  dual energy absorptiometry, MRI  =  magnetic resonance imaging, BIA  =  bioelectric impedance, WT  =  weight, WC  =  waist circumference, TC  =  total cholesterol, HDL  =  high density lipoprotein cholesterol, LDL  =  low density lipoprotein cholesterol, TG  =  triglycerides, NR  =  not reported, NA  =  not assessed.

**Table 2:** Intervention Characteristics.

Study	Completion Rate	Diet intervention	Diet compliance	Exercise intervention	Exercise compliance	Follow-up Intervention
**Long-term trials**						
Anderssen et al. (75)	D = 95% AE = 91% D+AE = 97% C = 100%	Diet counseling at baseline (participant and spouse), 3 and 9 mos.(participant only). Dietary advice was individualized according to participant dietary history and coronary heart disease risk factor profile. Energy restriction recommended for overweight participants. Level of energy restriction was not described.	Food-frequency questionnaire data indicated significantly greater reductions in energy intake in the D (−2268 kJ/day) and D+AE (−2003 kJ/day) compared with the AE (−497 kJ/day) and C groups (−589 kJ/day).	Aerobics, circuit training, and walk/jog. Group supervised format. 3 d/wk,60-80% peak HR, 60 min.	AE and D+AE groups attended 57% and 63% of scheduled sessions, respectively.	NA
Dengel et al. (81)	D = 58.8% D+AE = 62.2% C = 57.7%	Participants reduced energy intake by ∼500 kcal/day following an American Heart Association meal plan diet. Weekly behavioral sessions were held over the 10 mo. trial.	RD monitored adherence to the diet by analyzing weekly food records; however, food record data was NR.	Supervised stationary cycling and walking/jogging, both outdoors and on a treadmill, 3 days/wk., 75-80% max VO_2_, 40 min.	Significant increase in VO_2_ max in D+AE, not in D or C.	NA
Foster-Schubert et al. (76)	D = 89% AE = 91% D+AE = 92% C = 92%	Used modified versions of the dietary components of both the LookAhead and Diabetes Prevention trial. Total daily energy intake goals of 1,200 to 2,000 kcal/day (<30% fat) were prescribed based on baseline body weight. Weekly group behavioral counseling sessions were held during the first 6 mos. During mos. 7–12 participants attended 2 meetings/mo.; 1 meeting face-to-face and 1 meeting by phone or email.	No significant change in energy intake assessed by food-frequency questionnaire in any intervention group.	Treadmill, cycle ergometer or other aerobic machines, supervised on 3 of 5 days/wk. 70–85% max HR, 45 min.	AE and D+AE groups averaged 80% and 85% of the exercise goal of 225 min/wk., respectively.	NA
Pritchard et al. (78)	NR by group Overall = 88%	Personalized diet plan to achieve a negative energy balance of ∼500 kcal/day and included monthly behavioral counseling sessions	A combination of food records and recall data showed significantly decreased energy intake in the diet only group with no significant change in either the aerobic exercise or control groups.	Self-selected unsupervised activity. 71% walked/jogged. 3 days/wk, 65–75% max HR, 30 min.	Self-report exercise frequency = 3–7 sessions/wk. Significant increase in energy expenditure assessed by activity diary.	NA
Racette et al. (5)	D = 90% AE = 79% C = 77%	Reductions in energy intake of 16% over the initial 3 mos. and 20% over the remaining 9 mos. To educate participants on appropriate portion sizes, meals were provided by a metabolic kitchen over 5 consecutive days at week 3 and after the 3 mo. time point. Behavioral counseling sessions were held weekly for the first 6 mos. and less frequently thereafter	Average reduction in energy intake over the 12 mo. intervention based on doubly labeled water was ∼12%.	Choice of exercise at research facility, health club, home or outdoors. HR monitored all sessions. Frequency and duration prescribed to achieve 20% energy deficit comparable with deficit in D group.	Completed 5.8 sessions/wk. Average energy expenditure 317 kcal/session.	NA
Wood et al. (82)	D = 82% D+AE = 90% C = 91%	Diet, supervised by a registered dietician, that approximated the goals of the National Cholesterol Education Program Step 1 diet; i.e. ∼55% carbohydrate,30% fat, and dietary cholesterol below 300 mg/day. Group behavioral counseling sessions were conducted weekly during the first 3 mos., every other week for the next 3 mos., and monthly, thereafter.	Food record data food showed reduced energy intake in both men and women in the D and D+AE groups compared with C.	Supervised brisk walking and jogging, 3 day/wk., 60–80% max HR, 25 min/day initially, ramped to 45 min/day by study mo. 4.	Significant increase in VO_2_ max in D+AE, not in D or C.	NA
**Follow-up trials**						
Andreou et al.(83)	Weight loss D = 100% D+AE = 100% Follow-up D = 76% D+AE = 75%	Meal plan diet ∼1,500 kcal/day (50% carbohydrate, 30% fat, 20% protein). 6 behavioral sessions.	NR	Outdoor walking/jogging. Prescribed 3 or more days/wk., vigorous, 20 min/day, or 5 or more days/wk., moderate, 30 min/day or, 5 or more day/wk. any combination achieving at least 600 MET-min/wk. Unsupervised.	NR	No specific intervention during follow-up.
Hunter et al.(84)	Of 208 randomized, 121 (59%) met weight loss requirement (BMI 25 kg/m^2^). 97 of 121 (80% of those meeting weight loss requirement and 47% of those randomized complete follow-up testing.	Diet (∼800 kcal/day) food provided by metabolic kitchen. Energy restriction was maintained until BMI 25 kg/m^2^ was achieved.	NR	All exercise supervised	Compliance was required to remain in the study during the weight loss phase.	Exercise during follow-up was prescribed at 2 days/wk. Compliance not required to remain in the study. Divided sample into exercise adherers (≥60% of scheduled exercise sessions) and non-adherers (<60% of scheduled sessions) for analysis. Participants were provided instructions on eating a balanced diet focusing on low-density food.
				AE: 3 days/wk., 80% max HR, ramped from 20–40 min/session by wk. 8.		
				RE: ramped to 2 sets of 10 exercises, 80% 1-RM by wk. 4		
Melanson et al. 2004 (91)	Weight loss AE = 44.7% D+AE = 65.1% Follow-up AE = 40.4% D+AE = 51.2%	Energy reduced diet at ∼500 kcal/day below maintenance requirements. Included 2-4 meal replacements/day(bars, shakes, soups). Weekly behavioral counseling with a dietician and exercise professional	Data from food records indicated significantly greater reductions in energy intake in the D+AE (−621 kcal/day) compared with the AE group (−42 kcal/day)	Unsupervised outdoor walking, 5 days/wk. at a “moderate” pace. Duration individualized to achieve ∼300 kcal/session by wk. 4, increasing to 500 kcal/session by the end of the study	NR	Participants were asked to continue their assigned intervention (AE or D+AE) over follow-up period. Participants returned to the clinic every 2 wks. for assessment of resting HR, BP, and WT. Participants were not given instructions, advice or encouragement in regards to weight management.
Melanson et al. 2006 (92)	Weight loss AE = 84% D+AE(cereal) = 70% D+AE(no cereal) = 71% Follow-up AE = 82% D+AE(cereal) = 66% D+AE(no cereal) = 65%	Energy reduced diet at ∼500 kcal/day below maintenance requirements. The diet of one group contained a fiber-rich whole-grams cereal. During the weight loss phase, participants were asked to eat 2 meals/day containing cereal. Participants attended weekly behavioral counseling with an exercise professional and a registered dietician.	Energy intake from food records was 16.3%, 30% and 23% below baseline values in the AE, D+AE(cereal) and the D+AE(no cereal) groups, respectively	Unsupervised outdoor walking, 5 days/wk. at a “moderate” pace. Duration individualized to achieve ∼300 kcal/session by wk. 4, increasing to 500 kcal/session by the end of the study	NR	Participants asked to continue their assigned intervention (AE or D+AE) over follow-up period. Participants returned to the clinic every 2 wks. for assessment of resting HR, BP, and WT. Participants were not given instructions, advice or encouragement in regards to weight management. D+AE (cereal) group reduced cereal intake to 1 meal/day
Molenar et al.(85)	Weight lossD = 87%D+AE = 79%C = 53%Follow-upD = 76%D+AE = 75%C = 47%	Prescribed 7 individual sessions with dietician over 6 mos. Based on 3-day food records, meal plan prescribed to achieve and sustain a 5%–10% weight loss.	88% in D group and 89% in D+AE group visited dietician. D group completed average 4.5 sessions, D+AE group competed 4.1 sessions.	Prescribed 6 sessions with physiotherapist. Individualized attainable goals to increase daily activity were prescribed at session 1, support for activity provided at additional sessions.	87% of participants in the D+AE group visited the physiotherapist. Completed an average of 5.0 sessions.	No specific intervention during follow-up
Neumark-Sztainer et al. (86)	Weight lossD = 90%D+AE = 100%Follow-upD = 52%D+AE = 62%	Prescribed meal plan at ∼1,000 kcal/day with weekly behavioral counseling.	NR	Prescribed 15 min of running/walking, 5 times/wk. and 10 min of exercise 6 times/wk. Instruction and supervision provided for 1 hr./wk.	Participants exercised or walked 2–3 times/wk. for ∼15–20 min. Compliance by self-report was 34%.	Behavioral sessions every 2 wks. for 2 mos., and 1 per/wk. for 3 mos.
Skender et al.(77)	Weight lossD = 69%AE = 70%D+AE = 64%Follow-up D = 36% AE = 58% D+AE = 50%	Prescribed meal plan with 30% fat, 50% carbohydrate, and 20% protein designed not to exceed a weight loss of 1 kg/wkGroup behavioral counseling over 12 mos.: 12 wkly session, bi-weekly for 3 sessions, 3 monthly sessions	NR	Prescribed brisk walking, goal of 3-5 45-min sessions per wk at a perceived intensity that was vigorous but never strenuous	NR	No specific intervention during follow-up
Snel et al. (87)	Weight loss D = 100% D+AE = 100% Follow-up D = NR D+AE = NR	VLCD ∼450 kcal/day. Weekly behavioral counseling	100% of VLCD sachets provided were used	Minimum 4 days/wk. cycle ergometer at home, 30 min at 70% VO_2_ max, and 1 hr. in hospital training supervised by physiotherapist. Sessions monitored by HR	Home-averaged 5.3 sessions/wk. at 35.7 min/session. Participants attended 97% of in-hospital sessions	Regular diet ∼1,800 kcal/day. Resumed diabetes medication and usual care by their physician
Svendsen et al.(93)	Weight loss Overall = 98% Follow-up D = 92% D+AE+RE = 96% C = 76%	EI was restricted to 4.2 MJ/d (1.6 MJ/d formula diet +2.6 MJ/d freely chosen food).	EI from baseline to end of follow-up was reduced in both the D (−1079 kJ/day) and D+AE+RE group (−1471 kJ/day) and increased in C (+281 kJ/d).	Exercise was supervised and included 3–60 to 90 min sessions/wk. of combined AE (cycle ergometer at ≥70% VO_2_ max) and RE at ≥65% maximal lifting capacity.	From baseline to end of follow up V0_2_ max was reduced in C (−13.4%) and increased in D (+4%) and D+AE+RE (1%).	Participants were on their own but were encouraged to continue exercise and monitor EI to maintain weight loss
Van Aggel-Leijssen et al.(88)	Weight loss Overall = 93% Follow-up D = 75% D+AE = 70%	6-wks VLCD (2.1MJ). Wks 7-10 decreased formula diet increased food of choice. Wks. 7–8, 1.4MJ/d formula+3.5 MJ choice. Wks. 9–10, 0.7 MJ formula +4.9 MJ choice food Weekly meetings	NR	Cycle ergometer, outdoor walk/jog. 4 day/wk., 40% VO_2_ max, 60 min. Supervised 3 of 4 sessions/wk.	During follow-up, attended 57% of sessions. Estimated energy expenditure 335 kcal/session. No change in V0_2_ max.	Asked to maintain weight at post weight loss level. D+AE group asked to continue exercise.
Volpe et al.(79)	NR	EI designed to achieve weight loss of 0.5 to 1.0 kg/wk. Weekly meetings or 3 mos., bi-weekly meetings for 3 mos.	No change in EI assessed by food records over the weight loss period.	Supervised exercise on a Nordic Track. 5 days/wk, 30 min/session, intensity NR.	NR	Provided Nordic Tack for home use. Minimal contact with investigators.
						
Wadden et al.(89)	Weight loss NR Follow-up Overall = 78%	Diet was a 16 wk. VLCD (∼925 kcal/day) followed by a conventional energy restricted diet (1,200 to 1,500 kcal/day) for 31 wks. with behavioral counseling; weekly for 28 wks., bi-weekly for 20 wks.	AE groups were prescribed 3 supervised sessions step aerobics/wk. over the first 28 wks. and 2 supervised sessions/wk. over wks. 29–48.	AE groups were prescribed 3 supervised sessions step aerobics/wk. over the first 28 wks. and 2 supervised sessions/wk. over wks. 29–48.	55% of participants reported exercise in the follow-up period. Walking 2.8 times/wk., total of 13 min/wk.	No specific intervention during follow-up.
			RE was 3 supervised sessions of 10 exercises that progressed in resistance when more than 14 repetitions could be performed on 2 consecutive sets.	RE was 3 supervised sessions of 10 exercises that progressed in resistance when more than 14 repetitions could be performed on 2 consecutive sets		
Wang et al. (90)	Weight Loss NR Follow-up D = 73% D+AE: low intensity = 92% D+AE: high intensity = 85%	Food provided by metabolic kitchen. Energy deficit adjust to ∼11,760 kJ/wk.(∼1680 kJ/d). Deficits for D only from reduction in EI, D+AE groups deficit from decreased EI (∼10,080 kJ/wk., and EEEx(∼1680 kJ/wk).	Mean daily caloric intake was 100.1% of provided calorie level.	Supervised treadmill exercise 3 days/wk. Low intensity, 45–50% HRR, 55 min High intensity, 70–75% HRR, 30 min	Attended 85% of sessions. No change in VO_2_ max in any group.	No specific intervention during follow-up.
Wing et al. (80)	Weight loss D = 95% AE = 89% D+AE = 78% C = 80% Follow-up D = 95% AE = 84% D+AE = 80% C = 78%	EI of 800–1,000 kcal/day for the first 8 wks. of the intervention which was gradually adjusted to reach 1,200–1,500 kcal/day at wk. 16. Weekly behavioral counseling.	EI during the intervention was significantly lower in the D group compared with the AE, D+AE and C groups.	Increased PA to 1,500 kcal/wk. through brisk walking (3 miles/day). 2 supervised group walks available during wks. 1–10, 1 supervised walk thereafter.	At 6 mos. over 75% of participants achieved the 1,500 kcal/wk. goal.	Bi-weekly group meetings and 2–6-wk refresher courses in yr. 2.

Abbreviations: AE  =  aerobic exercise, RE  =  resistance exercise, C  =  control, D  =  diet, D+AE  =  diet plus aerobic exercise, D+RE  =  diet plus resistance exercise, D+AE+RE  =  diet plus aerobic plus resistance exercise, EI  =  energy intake, HR  =  heart rate, HRR  =  heart rate reserve, NR  =  not reported, NA  =  not assessed, VLCD  =  very low calorie diet.

### Diet vs. Aerobic Exercise

Seven trials, which represent 35% of the trials selected for this review, provided a comparison of long-term weight loss [5], [75]–[78], or weight loss with assessments after a period of follow-up [77], [79], [80] achieved by diet alone or aerobic exercise alone.

### Study Characteristics-Diet vs. Aerobic Exercise

#### Sample size

The median (range) sample size for diet groups with long-term comparison was 42 (18-118) and aerobic exercise groups was 24 (21-117) [5], [75]-[78]. The median (range) sample size for trials with a follow-up period was 37(15-42) for diet groups, and 24 (17-37) for aerobic exercise groups [77], [79], [80].

#### Trial duration

All trials with long-term comparisons included a period of active weight loss of 12 months [5], [75]–[78]. The length of the active weight loss and follow-up periods in the 3 trials with this design was variable. For example, Volpe et al [79] included a 6 month active intervention with a 6 month follow-up. Skender et al [77] included a 12 month active intervention with 12 month follow-up, while Wing et al [80] completed follow-up assessments 18 months after completing a 6 month active intervention.

#### Completion rate

Four trials with long-term comparisons provided completion rate by intervention group [5], [75]–[77] The median (range) completion for diet groups was 89.5% (69%–95%) and aerobic exercise groups was 85% (70%–91%). Pritchard et al [78] reported an overall completion rate of 88%. Two of the three trials that included assessments after a follow-up period reported completion rates [77], [80]. Skender et al [77] reported completion rates for the active intervention of 69% and 70% for the diet and aerobic exercise groups, respectively. The completion rate at follow-up was reduced to 36% and 58% for the diet and aerobic exercise groups, respectively. Wing et al [80] reported completion rates for the active intervention of 95% in the diet group and 89% in the aerobic exercise group and 95% for the diet group and 84% for the aerobic exercise group at follow-up.

#### Diet

All trials employed an energy restricted meal plan diet with behavioral counseling during the active intervention [5], [75]–[80].

#### Compliance with diet protocol

Four trials including long-term comparisons provided information on compliance with the prescribed diet [5], [75], [76], [78]. Anderssen et al [75] and Pritchard et al [78] both reported decreased energy intake in the diet but not the aerobic exercise groups. Energy intake was assessed by food-frequency questionnaire [75] or a combination of food records and recalls [78]. Racette et al [5] reported a 12% decrease in energy expenditure assessed by doubly labeled water over the 12 month intervention. Foster-Schubert et al [76] found no significant changes in energy intake assessed by food-frequency questionnaire in any intervention group. Volpe et al [79] found no change in energy intake assessed by food records in either the diet or aerobic exercise groups while Wing et al [80] reported significantly lower energy intake assessed by food-frequency questionnaire in the diet compared with the aerobic exercise group during the active intervention.

#### Aerobic exercise mode/supervision

A variety of exercise modes were employed including brisk walking/jogging [5], [75], [77], [78], [80], treadmill or cycle ergometer [5], [76], [78] and Nordic Track exercise machine [79]. Exercise was supervised in two trials, [75], [79], unsupervised in three trials [5], [77], [78] and partially supervised in two trials [76], [80].

#### Aerobic exercise frequency

Exercise was prescribed at 3 days per week in 2 trials [75], [78], 5 days/wk in three trials [76], [79], [80] and 3–5 days/wk in one trial [77]. Racette et al [5] individualized the number of weekly exercise sessions to achieve a target level of weekly exercise energy expenditure.

#### Aerobic exercise intensity

Exercise intensity was prescribed at between 60–85% of peak or maximum heart rate in three trials [75], [76], [78], and at a self-assessed “vigorous but never strenuous” level on one trial [77]. Exercise intensity was not specified in three trials [5], [79], [80].

#### Aerobic exercise duration

Five trials prescribed exercise duration by time; two trials prescribed 30 min/session [78], [79], two trials prescribed 45 min/session [76], [77], and one trial 60 min/session [75]. Racette et al [5] prescribed an exercise duration designed to achieve a 20% energy deficit designed to be comparable with the level of energy deficit prescribed for the diet group. Wing et al [80] prescribed walking at 3 miles/wk.

#### Compliance with exercise protocol

Five trials (5 of 7) provided data on compliance with the exercise intervention using a variety of metrics [5], [75], [76], [78], [80]. Anderssen et al [75] reported the aerobic exercise group attended 57% of scheduled exercise sessions. Participants in the Foster-Schubert [76] trial averaged 80% of the exercise goal of 225 min/wk., while Wing et al [80] reported 75% of participants achieved the prescribed exercise goal by the end of the active intervention. Pritchard et al [78] found that participants completed between 3–7 exercise session/wk., thus met their exercise goals. Participants in the Racette et al [5] trial completed and average of 5.8 exercise sessions/wk. with an average exercise energy expenditure of 317 kcal/session assessed by heart rate/oxygen consumption regression.

#### Intervention during follow-up

Three trials which compared diet with aerobic exercise included assessments after a follow-up period [77], [79], [80]. Volpe et al [79] provided participants with a Nordic Track exercise machine for home use during the follow-up period and had minimal contact with participants. Wing et al [80] provided bi-weekly group meetings over the first 6 months and two 6-week refresher courses during the final year of follow-up. Skender et al [77] provided no specific intervention during the follow-up period.

#### Outcomes assessed

In addition to body weight, two of the five trials with long-term comparisons assessed waist circumference [75], [76], three assessed body composition by DXA [5], [76], [78] and one trial assessed blood pressure, total, HDL, and LDL cholesterol and triglycerides [75]. One trial with assessments after a follow-up period assessed waist circumference and body composition by under-water weight [79] 2 trials assessed blood pressure, total, HDL, and LDL cholesterol and triglycerides [79], [80] while one trial assessed HbA_1c_, glucose and insulin [80].

### Participant characteristics - Diet vs. Aerobic Exercise

#### Age

Three of the five trials with long-term comparisons reported participant age by intervention group [5], [76], [78]. The median (range) age for diet groups was 55.6 yrs. (42.3–58.1 yrs.) aerobic exercise groups was 58.1 yrs. (44.9–58.8 yrs.) [5], [76], [78]. In the long-term trial by Anderssen et al [75] the mean age of the total sample was 40 yrs., while the age range in the trial by Skender et al [77] which provided both long-term and follow-up data was 25–45 yrs. Volpe et al [79] reported a mean age of 44.3 and 43.2 yrs. in the diet and aerobic exercise groups, respectively while the mean age in the trial by Wing et al [80] was 45.0 and 46.4 yrs, respectively.

#### Sex

One of the five trials with long-term comparisons included only women [76] and one trial included only men [78], while three trials included both men and women. The median (range) percent of women in these trials was 47% (9.6%–62%) [5], [75], [77]. The median (range) percent women in trials with assessments after a follow-up period was 51% (47%–79%) [77], [79], [80]. Only Volpe et al presented separate results for men and women [79].

#### BMI

Four of the five trials with long-term comparisons reported baseline BMI by intervention group ([5], [75], [76], [78]. The median (range) BMI for diet groups was 29.3 kg/m^2^ (27.2–31.1 kg/m^2^) and aerobic exercise groups was 28.9 kg/m^2^ (27.2–30.7 kg/m^2^). Two of the three trials with assessments after a follow-up period reported baseline BMI. BMI was 30.9 kg/m^2^ [79] and 36.1 kg/m^2^ [80] in the diet groups and 30.5 kg/m^2^ [79] and 36 kg/m^2^ [80] in the aerobic exercise groups in these trials.

#### Minority representation’

Two of the five trials with long-term comparisons provided information regarding minority participation [5], [76]. Foster-Schubert et al [76] included 15% minorities while minority representation was 10.2% in the trial by Racette et al [5]. None of the trials which included assessments after a follow-up period provided information on minority participation.

#### Physical activity level

Four of the five trials with long-term comparison that provided information on baseline level of physical activity recruited sedentary participants [5], [75]–[77]. Two of the three trials with assessments after a follow-up period that included information on baseline physical activity also recruited sedentary participants [77], [79].

#### Health status

With the exception of the trial by Anderssen et al [75] all trials with long-term comparisons recruited apparently healthy individuals. Anderssen et al [75] recruited participants with high diastolic blood pressure, high total cholesterol and triglycerides, and low HDL cholesterol. Trials with assessments after a follow-up period recruited healthy participants [77], [79], [80]; however; participants recruited by Wing et al [80] had one or two biological parents with DM2.

## Results-Diet vs. Aerobic Exercise

### Long term trials

Results for all trials with a diet vs. aerobic exercise comparison are presented in Table 3.

**Table 3:** Comparison of change in weight, body composition, and chronic disease risk factors in response diet compared with aerobic exercise.

Study	Variable	Baseline - end of active intervention		Baseline -end follow-up		During follow-up	
		D	AE	D	AE	D	AE
**Long-term trials**							
Anderssen et al. (75)	WT (kg/%)	−4.0/−4.2	−0.9/−1.0 ^nr^	na	na	na	na
	WC (cm)	−3.7	−1.9 ^nr^	na	na	na	na
	TC (mmol/l)	−0.23	+0.20 ^nr^	na	na	na	na
	LDL-C(mmol/l)	−0.16	−0.13 ^nr^	na	na	na	na
	HDL-C(mmol/l)	+0.05	+0.04 ^nr^	na	na	na	na
	TG (mmol/l)	−0.23	−0.24 ^nr^	na	na	na	na
	SBP (mmHg)	−6.4	−2.2 ^nr^	na	na	na	na
	DBP (mmHg)	−3.4	−2.7 ^nr^	na	na	na	na
Foster-Schubert et al. (76)	WT (kg/%)	−7.1/−8.5	−2.0/-2.4 *	na	na	na	na
	WC (cm)	−4.5	−2.0 *	na	na	na	na
	Fat mass (kg)	−6.1	−2.1 *	na	na	na	na
	Fat-free mass (kg)	−0.8	+0.4 *	na	na	na	na
Pritchard et al.(78)	WT (kg/%)	−6.3/−7.2	−2.6/−3.0 *	na	na	na	na
	Fat mass (kg)	−3.5	−1.8 *	na	na	na	na
	Fat-free mass (kg)	−2.5	−0.6 *	na	na	na	na
Racette et al. (5)	WT (kg/%)	−8/−10.2	−6.4/−8.4 ^ns^	na	na	na	na
	Fat mass (kg)	−6.2	−5.5 ^ns^	na	na	na	na
	Fat-free mass (kg)	−1.7	−0.9 ^ns^	na	na	na	na
**Follow-up trials**							
Skender et al. (77)	WT (kg/%)	−6.8/−6.9	−2.9/−3.1^ns^	+0.9/0.9	−2.7/2.9 ^ns^	7.7/7.8	+0.2/0.2 ^nr^
Volpe et al. (79)	WT (kg)	Figures only					
Men	WC (cm)	0	−1.2 ^ns^	−0.1	−1.2 ^ns^	−0.1	0.00 ^nr^
	Fat mass (kg)	−0.9	−1.6 ^ns^	+0.2	+0.2 ^ns^	+1.1	+1.8 ^nr^
	Fat-free mass (kg)	−1	+0.3 ^ns^	−1.4	+0.2 ^ns^	−0.4	−0.1 ^nr^
	TC (mmol/l)	+0.25	−0.16 ^ns^	+0.09	−0.18 ^ns^	−0.16	−.02 ^nr^
	LDL-C (mmol/l)	+0.09	+0.08 ^ns^	+ 0.21	+0.37 ^ns^	+0.12	+0.29 ^nr^
	HDL-C (mmol/l)	−0.28	−0.24 ^ns^	−0.18	−0.13 ^ns^	+0.10	+0.11 ^nr^
	TG (mmol/l)	−0.19	−0.32 ^ns^	+0.07	−0.20 ^ns^	+0.26	+0.10 ^nr^
	SBP (mmHg)	−18	−15.3 ^ns^	−13.3	−14.3 ^ns^	+4.7	+1.0^nr^
	DBP (mmHg)	−8.4	−7.7 ^ns^	−9.3	−7.9 ^ns^	−0.9	−0.20 ^nr^
Women	WT (kg)	Figures only					
	WC (cm)	−1.1	−3.6 ^ns^	+0.9	−3.9 ^ns^	+2.0	−0.30 ^nr^
	Fat mass (kg)	−0.3	−0.5 ^ns^	−0.4	−1.0 ^ns^	−0.10	−0.50 ^nr^
	Fat-free mass (kg)	+0.3	+0.1 ^ns^	+2.1	+0.9 ^ns^	+1.8	+0.80 ^nr^
	TC (mmol/l)	−0.13	+0.6 ^ns^	+0.26	+0.3 ^ns^	+0.39	+0.30 ^nr^
	LDL-C (mmol/l)	+0.29	+0.24 ^ns^	+0.15	+0.44 ^ns^	−0.14	+0.20 ^nr^
	HDL-C (mmol/l)	−0.37	−0.21 ^ns^	−0.24	−0.13 ^ns^	+0.13	+0.80 ^nr^
	TG (mmol/l)	−0.9	−0.9 ^ns^	+0.1	+0.03 ^ns^	+1.0	+0.93 ^nr^
	SBP (mmHg)	−3.7	−0.9 ^ns^	−1.5	+3.0 ^ns^	+2.2	+3.9 ^nr^
	DBP (mmHg)	+1	+0.6 ^ns^	−2.6	+6.3 ^ns^	−3.6	+5.7^nr^
Wing et al. (80)	WT (kg/%)	−9.1/−9.1	−2.1/−2.1 ^nr^	−2.1/−2.1	+1.0/1.0 ^nr^	+7.0/7.0	+3.1/3.1 ^nr^
	TC (mmol/l)	−0.49	+0.12 ^nr^	−0.12	+0.33 ^nr^	+0.37	+0.21 ^nr^
	LDL-C (mmol/l)	−0.32	+0.03 ^nr^	−0.16	+0.22 ^nr^	+0.16	+0.19 ^nr^
	HDL-C (mmol/l)	−0.1	+0.02 ^nr^	+0.02	+0.05 ^nr^	+0.12	+0.03 ^nr^
	TG (mmol/l)	−0.3	+0.12 ^nr^	+0.19	+0.33 ^nr^	+0.49	+0.21 ^nr^
	Glucose (mmol/l)	−0.2	0.00 ^nr^	+0.3	+0.40 ^nr^	+0.50	+0.40 ^nr^
	Insulin (pmol/l)	−24	−4.0 ^nr^	+20.0	+43.0 ^nr^	+44.0	+47.0 ^nr^
	HbA_1c_ (%)	+0.1	+0.10 ^nr^	−0.10	−0.10 ^nr^	−0.20	−0.20 ^nr^
	SBP (mmHg)	−10.2	−2.4 ^nr^	−0.8	+0.9 ^nr^	+9.4	+3.3 ^nr^
	DBP (mmHg)	−6.2	−1.7 ^nr^	+3.0	+2.0 ^nr^	+9.2	+3.7 ^nr^

Abbreviations: D  =  diet, AE  =  aerobic exercise, WT  =  body weight, WC  =  waist circumference, TC  =  total cholesterol, LDL  =  low density lipoprotein cholesterol, HDL  =  high density lipoprotein cholesterol, TG  =  triglycerides, na  =  not assessed, ns  =  between group difference for change non- significant, nr  =  between group difference for change not reported.

*between group difference for change significant at p<0.05 or greater.

#### Body weight

The median (range) weight loss for the five trials that provided long-term comparisons was 7.2% (4.2–10.7%) for diet groups and 2.4% (0.3%–8.4%) for aerobic exercise groups [5], [75]–[78]. Weight loss was significantly greater in the diet compared with the aerobic exercise groups in two trials [76], [78], non-significant in two trials [5], [77] and statistical significance of the between group difference was not reported in one trial [75].

#### Waist circumference

Waist circumference was reduced by 3.7 cm in the diet group and 1.9 cm in the aerobic exercise group (significance not reported) in the one long-term trial reporting this variable [75].

#### Fat mass

Three long-term trials assessed change in fat mass [5], [76], [78]. The median (range) change in fat mass was −6.1 kg (−3.5 to −6.2 kg) in diet and −1.8 (−2.2 to −5.5 kg) aerobic exercise groups. The reduction in fat mass was significantly greater in diet than aerobic exercise groups in two of the three trials [76], [78]. However, Racette et al [5] reported no significant difference between the change in fat mass in the diet group (−6.2 kg) compared with the aerobic exercise group (−5.5 kg).

#### Fat-free mass

Three long-term trials reported change in fat-free mass [5], [76], [78]. The median (range) change in fat-free mass was −1.7 kg (−0.8 kg to −2.5 kg) in the diet group and −0.6 kg (−0.9 kg to +0.3 kg) in the aerobic exercise groups. The reduction in fat-free mass was significantly greater in the diet compared with the aerobic exercise group in two of the three trials [76], [78]. Racette et al [5] reported no significant differences for change in fat-free mass between the diet (−1.7 kg) and aerobic exercise (−0.9 kg) groups.

#### Chronic disease risk factors

One long-term trial assessed change in chronic disease risk factors[75]; however, the significance of the between group differences were not reported. Anderssen et al [75] reported similar reductions in total cholesterol (diet = −0.23 mmol/l; aerobic exercise = −0.24 mmol/l), LDL-cholesterol (diet = −0.16 mmol/l; aerobic exercise = −0.13 mmol/l) and triglycerides (−0.23 mmol/l; aerobic exercise = −0.24 mmol/l), and similar increases in HDL-cholesterol (diet = 0.05 mmol/l; aerobic exercise = 0.04 mmol/l) in both the diet and aerobic exercise groups. Systolic (diet = −6.4 mmHg; aerobic exercise = −2.2 mmHg) and diastolic blood pressure (diet = −3.4 mmHg; −2.7 mmHg) were reduced in both the diet and aerobic exercise group.

### Trials with follow-up assessments

#### Body weight

Three trials provided data on weight change following active weight loss and follow-up periods of 6 months [79], 12 months [77] and 18 months [80]. Skender et al [77] observed no significant difference in weight loss between the diet (−6.9% and aerobic exercise groups (−3.0%) at the completion of the active intervention; however, after a 12 month follow-up period with no active intervention the diet group regained 113% of lost weight while the aerobic exercise group regained only 7% of the initial weight loss. Wing et al [80] reported weight loss of 9.1% in the diet group and 2.1% in the aerobic exercise group following the active intervention that was reduced to a 2.1% weight loss in the diet group and 1% weight gain in the aerobic exercise group at the 18 month follow-up. Thus, the diet and aerobic exercise groups regained 77% and 148% of their lost weight, respectively. Statistical significance of between group changes was not presented. Volpe et al [79] presented weight data only in figures. Small changes in weight were observed both during the active intervention and follow-up periods, with no significant difference between the diet and aerobic exercise groups for either men or women.

#### Waist circumference

Volpe et al [79] reported small decreases in waist circumference in the diet (−0.6 cm) and aerobic exercise groups (−2.4 cm) on completion of the active intervention which were essentially maintained in the aerobic exercise group (−2.6 cm) but not in the diet group (+0.4 cm) at 18 months. Sex differences for change in waist circumference were not apparent [79].

#### Fat mass/fat free mass

One trial assessed fat and fat-free mass following active weight loss and after a follow-up period. Volpe et al [79] observed no significant between group difference for change in fat mass at the completion of the active intervention (diet = −0.6 kg, aerobic exercise = −1.1 kg) or from baseline to the end of the follow-up period (diet = −0.1 kg, aerobic exercise = −0.7 kg). Similar results were observed for change in fat-free mass. No significant between group differences for change in fat-free mass were observed during the active intervention (diet = −0.4 kg, aerobic exercise = +0.2 kg) or from baseline to the end of the follow-up period (diet = +0.4 kg, aerobic exercise = +0.6 kg). No sex differences for change in fat mass or fat-free mass were observed [79].

#### Chronic disease risk factors

Volpe et al [79] reported no significant between group differences for change in total, HDL or LDL cholesterol, triglycerides, diastolic or systolic blood pressure in either men or women following completion of the active intervention (6 months), or at follow-up (6 months). At completion of the active intervention (6 months), Wing et al [80] reported significant within group reductions in the diet group in total, HDL and LDL cholesterol, systolic and diastolic blood pressure, fasting insulin and fasting glucose. Triglycerides and HbA_1C_ were not changed significantly. There were no significant changes in any of these variables in the aerobic exercise group following completion of the active intervention. At follow-up (24 months) the within group change from baseline for the diet group in total, HDL and LDL cholesterol, triglycerides and systolic blood pressure, and fasting insulin were not statistically significant. In the diet group fasting glucose and diastolic blood pressure increased significantly while HbA_1C_ was significantly decreased from baseline. With the exception of within group increases in fasting glucose and total cholesterol, there were no significant changes from baseline to the end of follow-up in the aerobic exercise group in any of the chronic disease risk factors assessed. The significance of between group changes at the end of the active intervention or follow-up were not reported [80].

### Diet vs. Diet+Aerobic Exercise

Fifteen trials, which represent 75% of the trials selected for this review, provided a comparison of long-term weight loss [75]–[77], [81], [82] or weight loss with assessments after a period of follow-up achieved by diet alone or diet+aerobic exercise [77], [79], [80], [83]–[90]. Both study and participant characteristics are presented in Tables 1 and 2.

### Study characteristics – Diet vs. Diet+Aerobic Exercise

#### Sample size

The median (range) sample size for diet groups with long-term comparison was 67 (42-118) and diet+aerobic exercise groups was 65 (37-117) [75]-[77], [81], [82]. The median (range) sample size for diet groups in trials with follow-up assessments was 30 (13-95) and diet+aerobic exercise groups was 32 (14-111) [77], [79], [80], [83]-[90].

#### Trial duration

The duration of the active intervention in four of the five trials with long-term comparisons was 12 months [75]–[77], [82]. Dengel et al [81] used a 10 month active intervention. The median (range) duration of the active weight loss intervention in trials with follow-up assessments was 5.8 months (3–12 months). The median (range) duration of the follow-up periods were 8 months (3-18 months) following completion of the active intervention [77], [79], [80], [83]–[90].

#### Completion rate

The median (range) completion rate for long-term comparisons was 82% (59%–95%) for diet groups and 90% (62–97%) for diet+aerobic exercise groups [75]–[77], [81], [82]. The completion rate for the active intervention for trials with follow-up assessments was not reported in two trials [79], [89] and not reported by intervention group in trials by Hunter et al [84] and Van Aggel-Leijssen [88] where the completion rates for the total sample were 59% and 93%, respectively. The median (range) completion rate at the end of the active intervention for the seven trials reporting this information was 92.5% (69–100%) for diet groups and 89.5% (64–100%) for diet+aerobic exercise groups [77], [80], [83], [85]–[87], [90]. Completion rates at follow-up were not reported in two trials [79], [87], and not reported by intervention group in one trial where the overall completion rate at follow-up was 60% [89]. The median (range) completion rate at follow-up for the eight trials reporting this information was 75.5% (36–95%) for diet groups and 72.5% (50–80%) for diet+aerobic exercise groups [77], [80], [83]–[86], [88], [90].

#### Diet

All long-term trials employed an energy restricted meal plan diet with behavioral counseling during the active intervention [75]–[77], [81], [82]. Several trials with follow-up assessments also employed energy restricted meal plans with behavioral counseling during the active weight loss intervention [77], [79], [80], [83], [85], [86]. However, trials with follow-up assessments also provided meals from a metabolic kitchen [84], [90] or used a very low calorie diet with behavioral counseling during active weight loss [87]–[89].

#### Compliance with diet protocol

Three trials including long-term comparisons provided information on compliance with the prescribed diet [75], [76], [82]. Anderssen et al [75] using a food-frequency questionnaire and Wood et al [82] using food records both reported decreased energy intake in the diet and diet+aerobic exercise groups compared with control. Foster-Schubert et al [76] found no significant changes in energy intake assessed by food-frequency questionnaire in either the diet or diet+aerobic exercise groups which was attributed to the imprecision of this measure of energy intake. However, relative fat intake (% of total daily energy intake) decreased 18% and 20% in the diet and die+aerobic exercise groups, respectively. Five trials will follow-up assessments provided information on compliance with the dietary intervention during weight loss [79], [80], [85], [87], [90]. Molenar et al [85] reported that 88% of participants attended ∼4 of the 7 behavioral sessions prescribed by the intervention. Snel et al [87] reported that participants consumed 100% of VLCD sachets provided while Wang et al [90] reported participants mean energy intake assessed by food records was 100.1% of the calorie level from food provided by a metabolic kitchen. Wing et al [80] reported significantly lower energy intake assessed by food-frequency questionnaire in both diet and diet+aerobic exercise groups compared with control. Volpe et al [79] found no change in energy intake in diet or diet+aerobic exercise groups, assessed by 3-day food records, over the weight loss intervention.

#### Aerobic exercise mode/supervision

Modes employed in long-term trials included brisk walking/jogging [75], [77], [81], [82] and treadmill or cycle ergometer exercise [76], [81]. Exercise was supervised in three trials [75], [81], [82], unsupervised in one trial [77] and partially supervised in one trial [76]. Aerobic exercise modes in trials with follow-up assessments included outdoor walking/jogging [77], [80], [83], [86] treadmill walking/jogging [88], [90] cycle ergometer [87], [88], Nordic Track exercise machine [79] and step aerobics [89]. Exercise was supervised in four trials [79], [84], [89], [90], partially supervised in four trials [80], [86]–[88], and unsupervised in two trials [77], [83]. Molenar et al [85] provide no specific information regarding any aspect of the exercise intervention.

#### Aerobic exercise frequency

Exercise in long-term trials was prescribed at 3 days per week in 3 trials [75], [81], [82], 5 days/wk. in one trial [76] and 3–5 days/wk. in one trial [77]. Trials with follow-up assessments prescribed exercise at 3 days/wk. [80], [84], [89], [90] 4 days/wk. [87], [88], 5 days/wk. [79], [86] and 3–5 days/wk. [77], [83].

#### Aerobic exercise intensity

Exercise intensity in long-term trials was prescribed at between 60–85% of peak or maximum heart rate in three trials [75], [76], [82], 75–80% VO_2_ max in one trial [81], and at a self-assessed “vigorous but never strenuous” level on one trial [77]. Trials with follow-up assessments prescribed exercise intensity at 80% maximal heart rate [84], 40% [88] and 70% maximal oxygen uptake [87], and 45–50% or 70–75% heart-rate-reserve [90]. Trials described exercise intensity as “vigorous but not strenuous” [77] and “moderate” or “vigorous” [83]. Exercise intensity was not specified in four trials that included follow-up assessments [79], [80], [86], [89].

#### Aerobic exercise duration

One long-term trial prescribed 40 min/session [81], three trials prescribed 45 min/session [76], [77], [82] and one trial 60 min/session [75]. Seven trials with follow-up assessments prescribed exercise duration by time [77], [79], [84], [86]–[88], [90]. The median (range) exercise duration in these trials was 35 min (25–60 min). Wadden et al [89] prescribed exercise duration to expend 300–400 kcal/session. Wing et al [80] prescribed waking at 3 miles/day while exercise duration was not specified in the trial by Andreou et al [83].

#### Compliance with exercise protocol

Four of the five long-term trials provided data on compliance with the exercise intervention [75], [76], [81], [82]. Anderssen et al [75] reported the diet+aerobic exercise group attended 63% of scheduled exercise sessions. Participants in the diet+aerobic exercise group in the Foster-Schubert [76] trial averaged 85% of the exercise goal of 225 min/wk. Both Dengel et al [81] and Wood et al [82] reported significant increases in maximal oxygen consumption in the diet+aerobic exercise, but not in diet or control groups. Seven of the eleven trials with follow-up assessments provided information on compliance with the exercise intervention [80], [84]–[88], [90]. Hunter et al [84] compared trial outcomes between participants who adhered (82%) or did not adhere (18%) to the aerobic exercise protocol during the follow-up period. Participants in the trial by Neumark et al [86] competed approximately 50% of assigned exercise sessions while Snel et al [87] and Wang et al [90] reported participants attended 97% and 85% of supervised exercise sessions, respectively. Van Aggel-Leijssen et al [88] found no change in aerobic capacity in the diet+aerobic exercise group over the course of the active intervention. Moelanar et al [85] asked participants to attend 6 sessions with a physiotherapist and reported that 87% of participants attended an average of 5 sessions.

#### Intervention during follow-up

Five of the 11 trials that compared diet with diet+exercise included no specific intervention during the follow-up period [77], [83], [85], [89], [90]. Volpe et al [79] provided participants with a Nordic Track exercise machine for home use during the follow-up period and had minimal contact with participants. Wing et al [80] and Neumark-Sztainer et al [86] provided continued behavioral sessions at a reduced frequency as compared with the active weight loss intervention. Hunter et al [84] and Van Aggel-Leijssen et al [88] asked participants to continue with the intervention assigned during weight loss. Participants in the trial by Snel et al [87] resumed their medications for DM2 and received usual care from their physician.

#### Outcomes assessed

In addition to body weight, two of the five trials with long-term comparisons assessed waist circumference [75], [76], and three assessed body composition, two by underwater weight [81], [82] and one by DXA [76]. Two trials assessed blood pressure, total, HDL, and LDL cholesterol and triglycerides [75], [82]. Five trials with follow-up assessments assessed waist circumference [79], [83], [85]–[87] and 6 trials assessed body composition, three by underwater weight [79], [88], [89], two by bioelectric impedance [83], [87] and one by DXA and magnetic resonance imaging [84]. Three trials assessed blood lipids (total, HDL, LDL cholesterol and triglycerides), 2 of which also assessed blood pressure [79], [80] and glucose, insulin and HbA_1c_ [80], [87].

### Participant characteristics – Diet vs. Diet+Aerobic Exercise

#### Age

Two of the five trials with long-term comparisons reported participant age by intervention group [76], [81]. The mean age in the diet groups was 61.0 yrs. and 58.1 yrs. and diet+aerobic exercise groups was 57.1 and 58 yrs. in the trials of Dengel et al. [81]and Foster-Schubert et al. [76],respectively. In the long-term trial by Anderssen et al [75] the mean age of the total sample was 40 yrs., while the age range in the trial by Skender et al [77], which provided both long-term and follow-up data was 25–45 yrs. Wood et al [82] reported a total sample mean age of 40.3 yrs. for men and 39.1 yrs. for women. Eight of the 11 trials with follow-up assessments reported participant age by participant group [79], [80], [83]–[85], [87]–[89]. The median (range) age in the diet groups was 40.8 yrs (29–59 yrs.) and diet+aerobic exercise groups was 41.2 (31–56) yrs. Neumark-Stzainer et al [86] and Skender et al [77] reported participant age ranges of 25–50 yrs., and 25–40 yrs., respectively. The mean sample age in the trial by Wang et al [90] was 58.6 yrs.

#### Sex

One of the five trials with long-term comparisons included only women [76] and one trial included only men [81], while three trials included both men and women [75], [77], [82]. The median (range) percent of women in these trials was 47% (9.6%–49%) [75], [77], [82]. Five trials with follow-up assessments included only women [83], [84], [86], [89], [90],one trial included only men [88], while five trials included both men and women [77], [79], [80], [85], [87]. The median (range) percent of women in these five trials was 47% (40–79%). Only 1 long-term trial [82] and one trial with follow-up assessments [79] presented results for men and women separately.

#### BMI

Three of the five trials with long-term comparisons reported participant BMI by intervention group [75], [76], [81]. The median (range) BMI was 30.1 kg/m^2^ (29.5–31.1 kg/m^2^) for the diet groups and 30.1 kg/m^2^ (28.6–31.0 kg/m^2^) for the diet+aerobic exercise groups. Wood et al [82] reported a mean sample BMI of 30.7 kg/m^2^ for men and 27.9 kg/m^2^ for women while Skender et al [77] did not report baseline BMI. Seven of the 11 trials with follow-up assessments reported baseline BMI by intervention group [79], [83], [85], [87]–[90] one trial reported mean BMI for the complete sample [86] one trial reported baseline percent body fat [90] and two trials did not report baseline BMI [77], [84]. The median (range) BMI for diet groups was 32.0 kg/m^2^ (30.9–37.9 kg/m^2^) and diet+aerobic exercise groups was 32.6 kg/m^2^ (29.1–37.3 kg/m^2^). Neumark-Sztainer et al [86] reported a baseline BMI of 30.3 kg/m^2^ in the complete sample. Wang et al [90] recruited participants with baseline percent body fat of 43%, 42.3% and 43.1% in the diet, diet+low intensity aerobic exercise and diet+high intensity exercise groups, respectively.

#### Minority representation

The percent minority representation was presented in two of the five long-term trials. The sample of Dengel et al [81] included no minorities while the sample of Foster-Schubert et al [76] included 15% minorities. The median (range) percent of minority representation in the five trials with follow-up assessments that provided this information was 34% (0–70%) ([84], [86], [87], [89], [90].

#### Physical activity level

All five trials with long-term comparison recruited sedentary participants [75]–[77], [81], [82]. Two of the three trials with assessments after a follow-up period that included information on baseline physical activity also recruited sedentary participants [77], [79].

#### Health status

With the exception of the trial by Anderssen et al [75] all trials with long-term comparisons recruited apparently healthy individuals. Anderssen et al [75] recruited participants with high diastolic blood pressure, high total cholesterol and triglycerides, and low HDL cholesterol. Trials will assessments after a follow-up period recruited healthy participants with the exception of Snel et al [87] who recruited participants with DM2 who were insulin dependent and Wing et al [80] who recruited healthy participants with one or two biological parents with DM2.

## Results: Diet vs. Diet+Aerobic Exercise

### Long-term trials

Results for all trials with a diet vs. diet+aerobic exercise comparison are presented in Table 4.

**Table 4:** Comparison of change in weight, body composition, and chronic disease risk factors in response to diet compared with diet plus aerobic exercise.

Study	Variable	Baseline - end of active intervention		Baseline - end follow-up		During follow-up	
		D	D+AE	D	D+AE	D	D+AE
**Long-term trials**							
Anderssen et al. (75)	WT (kg/%)	−4/−4.2	−5.6/−6.2 ^nr^	na	na	na	na
	WC (cm)	−3.7	−5.7 ^nr^	na	na	na	na
	TC (mmol/l)	−0.23	−0.48 ^nr^	na	na	na	na
	LDL-C (mmol/l)	−0.18	−0.39 ^nr^	na	na	na	na
	HDL-C (mmol/l)	+0.05	+0.13 ^nr^	na	na	na	na
	TG (mmol/l)	−0.23	−0.58 ^nr^	na	na	na	na
	SBP (mmHg)	−6.4	−5.9 ^nr^	na	na	na	na
	DBP (mmHg)	−3.4	−5.2 ^nr^	na	na	na	na
Dengel et al. (81)	WT (kg/%)	−9.3/−10.1	−8.1/−8.6 ^ns^	na	na	na	na
	Fat mass (kg)	−6.8	−6.7 ^ns^	na	na	na	na
	Fat-free mass (kg)	−2.1	−1.3 ^ns^	na	na	na	na
Foster-Schubert et al. (76)	WT (%)	−7.1/−8.5	−8.9/−10.8 *	na	na	na	na
	WC (cm)	−4.5	−7.5 *	na	na	na	na
	Fat mass (kg)	−6.1	−8.2 *	na	na	na	na
	Fat-free mass (kg)	−0.8	−0.4 ^ns^	na	na	na	na
Wood et al. (82)	WT (kg/%)	−5.1/−5.2	−8.7/−8.8 *	na	na	na	na
Men	Fat mass (kg)	−4.3	−7.8 *	na	na	na	na
	TC (mmol/l)	−0.42	−0.38 ^ns^	na	na	na	na
	LDL-C (mmol/l)	−0.39	−.0.27 ^ns^	na	na	na	na
	HDL-C (mmol/l)	+0.02	+0.14 *	na	na	na	na
	TG (mmol/l)	−0.12	−0.48 *	na	na	na	na
	SBP (mmHg)	−4.1	−5.4 ^ns^	na	na	na	na
	DBP(mmHg)	−2.4	−4.9 ^ns^	na	na	na	na
Women	WT (%)	−4.1/−5.5	−5.1/−6.8 *	na	na	na	na
	Fat mass (kg)	−4.0	−5.5 ^ns^	na	na	na	na
	TC (mmol/l)	−0.39	−0.28 ^ns^	na	na	na	na
	LDL-C (mmol/l)	−0.28	−0.29 ^ns^	na	na	na	na
	HDL-C (mmol/l)	−0.15	+0.02 *	na	na	na	na
	TG (mmol/l)	+0.09	−0.02 ^ns^	na	na	na	na
	SBP (mmHg)	−4.1	−3.6 ^ns^	na	na	na	na
	DBP (mmHg)	−2.2	−2.0 ^ns^	na	na	na	na
**Follow-up trials**							
Andreou et al. (83)	WT (kg/%)	−6.1/−7.5	−15.5/−19.5 ^nr^	−8.4/−10.3	−17.3/−21.7 ^nr^	−2.3/−2.8	−1.8/−2.2 ^nr^
	WC (cm)	−5.5	−11.2 ^nr^	−8.0	−14.2 ^nr^	−2.5	−3.0 ^nr^
	%fat	−0.5	−8.2 ^nr^	−1.2	−10.0 ^nr^	−0.7	−1.8 ^nr^
Hunter et al.(84)	WT (kg/%)	na	na	na	na	+6.4/9.8	Adhere = 3.1/4.9% *
	WT(kg/%)	na	na	na	na	+6.4/9.8	Non-Adhere = 7.1/10.9 ^ns^
	WC (cm)	na	na	na	na	+5.5	Adhere = 1.9 ^ns^
	WC (cm)	na	na	na	na	+5.5	Non-Adhere = +7.9 ^ns^
	% Fat	na	na	na	na	+5.3	Adhere = 3.4 *
	% Fat	na	na	na	na	5.3	Non-Adhere = 6.4 ^ns^
	Visceral fat (cm^2^)	na	na	na	na	+12.4 **	Adhere = 0.8 ^ns^ within
	Visceral fat (cm^2^)	na	na	na	na	+12.4 **	Non-Adhere = 25.5 **
Molenar et al. (85)	WT (kg/%)	−2.2/−2.3	−3.0/−3.2 ^ns^	−2/−2.1	−3.1/−3.3 ^ns^	−0.2/−0.2	+0.1/0.1 ^nr^
	WC (cm)	−2.1	−3.7 ^ns^	−2.1	−4.2 ^ns^	0.0	−0.5 ^nr^
Neumark-Sztainer et al. (86)	WT (kg/%^a^)	−3.8	−3.6 ^ns^	−3.9	−4.6 ^ns^	−0.1	−1.0 ^nr^
	WC (cm)	−7.4	−8.5 ^ns^	na	na	na	na
Skender et al. (77)	WT (kg/%)	− 6.8/−6.9	−8.9/−8.9 ^ns^	+0.9/0.9	−2.2/−2.2%	+7.7/7.8 ^ns^	+6.7/6.7 ^nr^
Snel et al. (87)	WT (kg/%)	−24/−21.2	−28.0/−24.6 *	−10/−8.8	−16.0/−14.0 *	14/12.4	+12/12.2 ^nr^
	WC (cm)	−19	−25.0 *	−8	−16.0 *	+11	+9.0 ^nr^
	Fat mass (kg)	−16.7	−21.9 ^nr^	−5.7	−10.0 ^nr^	+11.0	+11.9 ^nr^
	TC (mmol/l)	−0.6	−0.9 ^nr^	−0.6	−1.1 ^nr^	0.0	−0.2 ^nr^
	LDL-C (mmol/l)	−0.7	−0.6 ^ns^	−1.1	−0.9 ^ns^	−0.2	−0.3 ^nr^
	HDL-C (mmol/l)	0	+0.1 ^ns^	+0.2	0.0^ns^	+0.2	+0.1^nr^
	TG (mmol/l)	−0.8	−1.3 ^ns^	+0.5	−0.6 ^ns^	+0.2	+0.7^nr^
	Glucose (mmol/l)	−4.4	−4.3 ^ns^	+1.0	−1.7 ^ns^	+5.4	+2.6 ^nr^
	Insulin (mU/l)	−11	−16.0 *	−7	−12.0 *	+4	+4.0 ^nr^
	HbA_1c_ (%)	−1.1	−1.5 ^ns^	+0.4	−0.3^ns^	+1.5	+1.2 ^nr^
	SBP (mmHg)	−21	−13.0 ^ns^	−4.0	+1.0 ^ns^	+17	+14 ^nr^
	DBP (mmHg)	−9	−6.0 ^ns^	+2.0	+1.0 ^ns^	+11.0	+7.0 ^nr^
Van Aggel-Leijssen et al.(88)	WT (kg/%)	−15.4/−14.9	−14.8/−14.4 ^ns^	−8.3/−8.0	−5.9/−5.8 ^ns^	7.1/6.9	+8.9/8.6 ^nr^
	Fat mass (kg)	−12.6	−12.5^ns^	−7.9	−6.7 ns	+4.7	+5.8 ^nr^
	Fat-free mass (kg)	−2.1	−2.1 ^ns^	−0.3	−0.8 _ns_	+1.8	+1.3 ^nr^
Volpe et al. (79)	WT (kg)	Figures only					
Men	WC (cm)	0	−4.9 *	−0.1	−1.9 ^ns^	−0.1	+3.0 ^nr^
	Fat mass (kg)	−0.9	−4.5 ^ns^	+0.2	−2.8 ^ns^	+1.1	+1.7 ^nr^
	Fat-free mass (kg)	−1	−0.3 ^ns^	−1.4	+0.1 ^ns^	−0.4	+0.4 ^nr^
	TC (mmol/l)	0.25	−0.27 ^ns^	+0.09	−0.04 ^ns^	−0.16	+0.11 ^nr^
	LDL-C (mmol/l)	0.09	+ 0.02 ^ns^	+ 0.21	+ 0.25 ^ns^	+0.12	+0.23 ^nr^
	HDL-C (mmol/l)	−0.28	−0.22 ^ns^	−0.18	−0.13^ns^	+0.10	+0.90 ^nr^
	TG (mmol/l)	−0.19	−0.24 ^ns^	+0.07	−0.20 ^ns^	+0.26	+0.04 ^nr^
	SBP (mmHg)	−18	−11.8 ^ns^	−13.3	−15.6 ^ns^	+4.7	−3.8 ^nr^
	DBP (mmHg)	−8.4	−9.7 ^ns^	−9.3	−12.3 ^ns^	−0.9	− 2.6 ^nr^
Women	WT (kg)	Figures only					
	WC (cm)	−1.1	+0.1 ^ns^	+0.9	−2.1^ns^	+2	−2.2 ^nr^
	Fat mass (kg)	−0.3	−4.2 *	−0.4	−1.9 ^ns^	−0.1	+2.3 ^nr^
	Fat-free mass (kg)	+0.3	+1.5 ^ns^	+1.2	+1.1 ^ns^	+0.9	−0.4 ^nr^
	TC (mmol/l)	−0.13	−0.7 ^ns^	+0.26	−0.21 ^ns^	+0.39	−0.14 ^nr^
	LDL-C (mmol/l)	0.29	+0.38 ^ns^	+0.15	−0.10 ^ns^	−0.14	−0.24 ^nr^
	HDL-C (mmol/l)	−0.37	−0.26 ^ns^	−0.24	−0.23 ^ns^	+0.13	+0.03 ^nr^
	TG (mmol/l)	−0.9	−0.34 ^ns^	+0.1	−0.26 ^ns^	+1.0	+0.08 ^nr^
	SBP (mmHg)	−3.7	−4.3 ^ns^	−1.5	+1.2 ^ns^	+2.2	+5.5 ^nr^
	DBP (mmHg)	+1	−4.3 ^ns^	−2.6	+1.2 ^ns^	−3.6	+5.5 ^nr^
Wadden et al. (89)	WT (kg/%)	−15.3/−16.3	−13.5/−13.9 ^ns^	−6.9/−7.7	−8.5/−8.6 ^ns^	+8.4/8.6	+5.0/5.3 ^nr^
Wang et al. (90)	WT (kg/%)	−12.8/−14	LI = −13.1/−14.8 ^ns^	−8.1/−8.0	LI = −6.8/−7.7 ^ns^	+4.7/6	+6.3/7.1 ^nr^
		−12.8/14	HI = −10.8/−11.0^ns^	−8.1/−8.0	HI = −7.0/−7.8 ^ns^	+5.8/6.2	+3.1/3.2 ^nr^
Wing et al. (80)	WT (kg/%)	−9.1/−9.1	−10.3/−10.4 ^nr^	−2.1/−2.1	−2.1/−2.5 ^nr^	+7.0/7.0	+8.2/7.9 ^nr^
	TC (mmol/l)	−0.49	−0.33 ^nr^	−0.12	+0.09 ^nr^	+0.37	+0.42 ^nr^
	LDL-C (mmol/l)	−0.32	−0.13 ^nr^	−0.16	+0.12 ^nr^	+0.16	+0.25 ^nr^
	HDL-C (mmol/l)	−0.1	−0.06 ^nr^	+0.02	+0.05 ^nr^	+0.12	+0.11 ^nr^
	TG (mmol/l)	−0.3	−0.69 ^nr^	+0.19	−0.28 ^nr^	+0.21	+0.41 ^nr^
	Glucose (mmol/l)	−0.2	−0.20 ^nr^	+0.30	+0.50 ^nr^	+0.50	+0.70 ^nr^
	Insulin (pmol/l)	−24	−37.0 ^nr^	+20.0	−12.0 ^nr^	+44.0	+25.0 ^nr^
	HbA_1c_ (%)	0.1	+0.03 ^nr^	−0.10	+0.04 ^nr^	−0.20	+0.01 ^nr^
	SBP (mmHg)	−10.2	−12.3 ^nr^	−0.8	−4.8 ^nr^	+9.4	+7.5 ^nr^
	DBP (mmHg)	−6.2	−6.9 ^nr^	+3.0	−0.2 ^nr^	+9.2	+6.7 ^nr^

Abbreviations: D  =  diet, D+AE  =  diet plus aerobic exercise, WT  =  body weight, WC  =  waist circumference, TC  =  total cholesterol, LDL  =  low density lipoprotein cholesterol, HDL  =  high density lipoprotein cholesterol, TG  =  triglycerides, na  =  not assessed, ns  =  between group difference for change non- significant, nr  =  between group difference for change not reported.

a baseline weight not reported.

*between group difference for change significant at p <0.05 or greater.

**within group change significant at p <0.05 or greater.

#### Body weight

The median (range) for weight loss was 6.9% (4.2–10.1%) in diet groups and 8.8% (6.2–11.5%) in diet+aerobic exercise groups. Weight loss was greater in the diet+aerobic exercise group compared with the diet only group in four of five trials. However, between group differences were statistically significant in only two [76], [82] of the four trials providing this information. Wood et al [82] reported significantly greater weight loss in the diet+aerobic exercise group compared with the diet group in both men and women.

#### Waist circumference

Two long-term trials assessed change in waist circumference. Anderssen et al [75] reported decreased waist circumference in both the diet (−3.7 cm) and diet+aerobic exercise groups (−5.7 cm); however statistical significant of the between group difference was not reported. Foster-Schubert et al [76] reported significantly greater reductions in waist circumference in the diet+aerobic exercise (−7.0 cm) compared with the diet only group (−4.5 cm).

#### Fat mass/fat-free mass

Fat mass was assessed in three long-term trials [76], [81], [82]. Dengel et al [81] found no significant difference in change in fat mass between diet (−2.1 kg) and diet+aerobic exercise (−1.3 kg) groups. However, both Foster-Schubert et al [76] (diet = −6.1 kg; diet+aerobic exercise = −8.2 kg) and Wood et al [82] in men (diet = −4.3 kg; diet+aerobic exercise = −7.8 kg) observed significantly greater loss of fat mass in diet+aerobic exercise compared with diet only groups. Wood et al [82] observed no significant between group difference for change in fat mass in women (diet = −4.0 kg, diet+aerobic exercise = −5.5 kg). Dengel et al [81] reported decreased fat-free mass in both the diet (−2.1 kg) and diet+aerobic exercise groups (−1.3 kg) with no significant between group difference. Foster-Schubert et al [76] also reported decreased fat-free mass in both the diet (−0.8 kg) and diet+aerobic exercise groups (−0.4 kg) with no significant between group difference. Wood et al [82] did not present data on fat-free mass.

#### Chronic disease risk factors

Two long-term trials compared change in chronic disease risk factors between diet and diet+aerobic exercise groups [75], [82]. Anderssen et al [75] reported decreases in both systolic (diet = −6.4 mmHg; diet+aerobic exercise = −5.9 mmHg) and diastolic blood pressure (diet = −3.4 mmHg; diet+aerobic exercise = −5.2 mmHg) in diet and diet+aerobic exercise groups. The statistical significance of the between group differences was not reported. Wood et al [82] reported decreased systolic and diastolic blood pressure in both diet and diet+aerobic exercise groups. The difference for change between groups was not significant. Anderssen et al [75] reported larger decreases in total cholesterol (diet = −0.23 mmol/l, diet+aerobic exercise = −0.48 mmol), LDL cholesterol (diet = −0.18 mmol/l; diet+aerobic exercise = −0.39 mmol/l) and trigylcerides (diet = −0.23 mmol/l; diet+aerobic exercise = −0.58 mmol/l) and larger increases in HDL cholesterol (diet = +0.5 mmol/l; diet+aerobic exercise = +0.13 mmol/l) in the diet+exercise compared to the diet group; however, statistical significance of the between group differences was not reported. Wood et al [82] found no significant difference for change in either total or LDL cholesterol between diet and diet+aerobic exercise groups for either men or women. The increase in HDL cholesterol was significantly greater in the diet+aerobic exercise groups (men = +0.14 mmol/l; women = +0.2 mmol/l) compared with the diet group (men = +0.2 mmol/l; women = −0.15 mmol/l) in both men and women. Among men, the decrease in triglycerides was significantly greater in the diet+aerobic exercise group (−0.48 mmol/l) compared with the diet group (−0.12 mmol). No between group differences were shown for change in triglycerides in women (diet = +0.9 mmol; diet+aerobic exercise = −0.2 mmol/l).

### Trials with follow-up assessments

#### Body weight

Eight of the 11 trials that assessed weight change, both after an active intervention and a follow-up period, reported or provided numerical data with which percent weight change could be calculated [77], [80], [83], [85], [87]–[90]. The median (range) for weight loss from baseline to completion of the weight loss intervention in these 8 trials was 11.6% (2.3–21.2%) in diet groups and 13.6% (3.2–24.6%) in diet+aerobic exercise groups. Weight loss was greater in diet+aerobic exercise compared with diet only groups in six trials [77], [79], [80], [83], [85], [87]; however, the between group differences were statistically significant in only one trial, in women but not men [79]. Six of these 8 trials reported clinically significant (≥5%) weight loss at the completion of the active intervention in both the diet and diet+aerobic exercise groups [77], [80], [87]–[90].

The median (range) percent weight loss from baseline to the completion of follow-up was 7.9% (+0.9 to −10.3%) in diet groups and 6.8% (−2.5 to −21.7%) in diet+aerobic exercise groups. Weight loss was greater in the diet+aerobic exercise compared with diet only groups in 7 of 9 trials providing this data [77], [79], [80], [83], [85]–[87], [89], [90]; however, between group differences were not statistically significant in the 7 trials that reported the significance for between group differences [77], [79], [85]–[87], [89], [90]. Weight change from baseline to the completion of follow-up did not differ by sex in the one follow-up trial reporting this data [79].

Four of the 6 trials that reported ≥5% weight loss in both the diet and diet+aerobic exercise groups at the end of the active intervention continued to observe ≥5% weight loss in both groups at follow-up [87]–[90]. Among the 6 trials reporting clinically significant weight loss on completion of the weight loss intervention, the median (range) of percent of lost weight regained at follow-up was 57.5% (37–113%) for diet groups and 52.5% (35–75%) for diet+aerobic exercise groups [77], [80], [87]–[90].

Hunter et al [84] reassessed body weight in a sample of initially overweight and obese women one-year following completion of a weight loss intervention by diet (800 kcal/day,food from metabolic kitchen) or diet+aerobic exercise. During weight loss, supervised aerobic exercise (treadmill) was prescribed at 3 days/week and ramped to 40 min/session at 80% maximal heart rate by week eight. Compliance with the exercise protocol was required to remain in the study. The weight loss intervention was continued until participants reached a BMI of 25 kg/m^2^ (mean 154 days) at which time they were asked to continue with their original group assignment with supervised exercise frequency reduced to two days/week for exercise groups over the next 12 months. All groups were given instruction on following a balanced diet that focused on consumption of low-density foods. Exercise during the follow-up period was not required to remain in the study, thus two groups were formed; participants who adhered (≥60% of scheduled sessions) and those who did not adhere (<60% of scheduled sessions). Results indicated that body weight increased significantly during the one-year follow-up, with a significant group by time interaction. The increase in weight from the end of weight loss was significantly less in participants who adhered to aerobic exercise (+4.9%) compared with those who failed to adhere (+10.9%) or in the diet group (+9.9%).

#### Waist circumference

Four trials with follow up assessment reported change in waist circumference [79], [83], [85], [87]. The median (range) change in waist circumference from baseline to the end of the active intervention was −2.1 cm (0 to −19 cm) for diet groups and −4.9 cm (+0.1 to −25 cm) for diet+aerobic exercise groups. Volpe et al [79] reported statistically significant between group differences for change for waist circumference, in men (diet = 0 cm; diet+aerobic exercise = −4.9 cm) but not in women (diet = +0.1, diet+aerobic exercise = +0.9 cm). Andreou et al [83] reported larger decreases in waist circumference in the diet+aerobic exercise (−11.2 cm) compared to the diet group (−5.5 cm); however, the statistical significance of the between group difference was not reported.

The median (range) change in waist circumference from baseline to the completion of follow-up was −2.1 cm (+0.9 cm to −8.0 cm) for diet groups and −4.2 cm (−1.9 to −16.0 cm) in diet+aerobic exercise groups [79], [83], [85], [87]. No significant between group differences were noted. Andreou et al [83] reported larger decreases in waist circumference in the diet+aerobic exercise (−14.2 cm) compared to the diet group (−8.0 cm). The statistical significance of the between group difference was not reported.

Waist circumference was unchanged or slightly decreased during the follow-up period in two trials ([83], [85] and increased in two trials [79], [87]. Participants in the trial by Snel et al [87] regained a large portion of the losses in waist circumference over the follow-up period (diet = 58%; diet+aerobic exercise  = 36%). Volpe et al [79] reported that men in the diet+aerobic exericise group who had a significant reduction in waist circumference during the active intervention (−4.9 cm) regained 61% of the loss during follow-up. Hunter et al [84], using the previously described study protocol, found that waist circumference increased significantly during a one-year follow-up, with a significant group by time interaction. The increase in waist circumference from the end of weight loss was significantly less in participants who adhered to aerobic exercise (+1.9 cm) compared with those who failed to adhere (+7.9 cm) or in the diet group (+5.5 cm).

#### Fat mass/% fat

Four trials with follow-up assessments reported change in fat mass or % fat over the active intervention [79], [83], [87], [88]. The median (range) change in fat mass was −13.2 kg (−0.6 to −16.7 kg) for the diet groups and −12.7 kg (−4.3 to −21.8 kg) for diet+aerobic exercise groups [79], [87], [88]. Between group differences for change in fat mass were statistically significant only in women in the trial reported by Volpe et al [79] (diet = −0.3 kg; diet+aerobic exercise = −4.2 kg). Andreou et al [83] reported greater loss of percent body fat in the diet+aerobic exercise group (−8.2%) compared with the diet group (−0.5%); however statistical significance of the between group difference was not reported.

The median (range) change in fat mass from baseline to the completion of follow-up was −5.7 kg (−0.1 kg to −7.9 kg) for diet groups and −6.7 kg (−2.4 to −10 kg) in the diet+aerobic exercise groups [79], [87], [88]. Andreou et al [83] reported significantly greater loss of percent body fat in the diet+aerobic exercise group (−10.0%) compared with the diet group (−1.2%).

Fat mass increased in both diet and diet+aerobic exercise groups during the follow-up period in three trials [79], [87], [88]; however Andreou et al [83] showed small continued decreases in percent body fat (diet = −0.7%; diet+aerobic exercise = −1.8%) during follow-up. Snel et al [87] reported participants regained 66% and 54% of their fat mass loss in the diet and diet+aerobic exercise groups, respectively over a follow-up of 14 months. Van Aggel-Leijssen [88] reported participants in the diet group regained 40% of their fat mass loss while participants in the diet+aerobic exercise group regained 47% of lost fat mass over 40 wks. Volpe et al [79] showed significant loss of fat mass during the active intervention only in the diet+aerobic exercise group. Over the follow-up period (6 months) men in the diet+aerobic exercise group regained 38% of fat mass loss while women regained 55% of their fat mass loss. Hunter et al [84],using the previously described study protocol, found that the increase in percent body fat from the end of weight loss was significantly less in participants who adhered to aerobic exercise (+3.4%) compared with those who failed to adhere (+6.4%) or in the diet group (+5.3%). Intra-abdominal adipose tissue increased significantly from the end of weight loss in both the diet group (+24.8%) and the group non-adherent to the aerobic exercise protocol (+54.4%) but not in the group who adhered to aerobic exercise (1.6%).

#### Fat-free mass/%lean mass

There were no significant between group differences for change in fat-free mass from baseline to completion of the weight loss intervention or from baseline to the end of follow-up in the two trials that reported this outcome [79], [88]. Andreou et al [83] showed a non-significant increase in percent lean body mass in the diet only group (+0.5%) and a significant increase in percent lean body mass in the diet+aerobic exercise group (8.2%) over the active intervention (18 wks.). At follow-up, 18 weeks after completing the active intervention, Andreou et al [83] found a significant between group difference in change from baseline for percent lean body mass which was reduced in the diet group (−1.1%) and increased in the diet+aerobic exercise group (+10%). Volpe et al [79] reported no significant difference for change in fat-free mass between diet and diet+aerobic exercise groups following completion of the weight loss intervention or from baseline to the end of follow-up in either men or women.

#### Chronic Disease Risk Factors

Three trials reported chronic disease risk factors [79], [80], [87]. At completion of the active intervention (6 months), Wing et al [80] reported significant within group reductions in total, HDL and LDL cholesterol, systolic and diastolic blood pressure, fasting insulin and fasting glucose in the diet only group. Triglycerides and HbA_1C_ were not changed significantly. In the diet+aerobic exercise group there were significant reductions in total cholesterol, triglycerides, systolic blood and diastolic blood pressure, triglycerides, glucose, and insulin. HDL cholesterol, LDL cholesterol and HbA_1C_ were not significantly changed. At follow-up (24 months) the within group change from baseline for the diet group in total, HDL and LDL cholesterol, triglycerides and systolic blood pressure, and fasting insulin were not statistically significant. In the diet group fasting glucose and diastolic blood pressure increased significantly while HbA_1C_ was significantly decreased from baseline. For the diet+aerobic exercise group, there were no significant within group changes from baseline in total, HDL and LDL cholesterol, triglycerides, systolic and diastolic blood pressure, and fasting insulin. Both glucose and fasting insulin were significantly increased from baseline. Thus, risk factor changes associated with the active intervention were not maintained at follow-up. The statistical significance of between group changes at the end of the active intervention or follow-up were not reported [80]. Snel et al [87] in a sample of obese patients with insulin dependent DM2 reported that significant reductions in systolic and diastolic blood pressure, triglycerides, LDL cholesterol, glucose and HbA_1c_ observed in the diet only group following completion of the active intervention (4 months) were no longer significant at follow-up (18 months). In the diet group, insulin was significantly reduced at both 4 and 18 months, HDL cholesterol was significantly increased only at 18 months, while total cholesterol was not significantly altered at either 4 or 18 months. In the diet+aerobic exercise group, significant reductions in systolic and diastolic blood pressure, triglycerides, glucose and HbA_1c_ observed at 4 month were no longer significant at 18 months. Insulin, total and LDL cholesterol were significantly reduced from baseline at both 4 and 18 months, while HDL cholesterol was significantly increased only at 18 months. Volpe et al [79] reported no significant changes in systolic blood or diastolic blood pressure, total, HDL or LDL cholesterol, or triglycerides in either the diet or diet+aerobic exercise groups after completion of the active intervention (6 months) or at follow-up (12 months) in either men or women.

### Aerobic Exercise vs. Diet+Aerobic Exercise

Seven trials, which represent 35% of the trials selected for this review, provided a comparison of long-term weight loss [75]–[77] or weight loss with assessments after a period of follow-up achieved by aerobic exercise or diet+aerobic exercise [77], [79], [80], [91], [92]. The trial by Skender et al [77] provides data for both a long-term intervention (12 months) and a follow-up (12 months). Study and participant characteristics are presented in Tables 1 and 2.

### Study characteristics

#### Sample size

In long-term trials, Anderssen et al [75] included 49 and 65 participants in the aerobic and diet+aerobic exercise groups, respectively, while Foster-Schubert et al [76] included 117 participants in both the aerobic exercise and diet+aerobic exercise groups. The median (range) sample size for the five trials that included follow-up assessments was 43 (34–47) for aerobic exercise groups and 42 (28–46) for diet+aerobic exercise groups [77], [79], [80], [84], [91], [92].

#### Trial Duration

Both long-term trials were 12 month in duration [75], [76]. Two trials with follow-up assessments used 12 week active interventions with 12 week follow-up periods [91], [92]. Both Volpe et al [79] and Wing et al [80] used 6 month active interventions. Volpe et al [79] reassessed participants after 6 months while Wing et al [80] completed follow-up assessments 18 months after completion of the active intervention. Skender et al [77] assessed participants after a 12 month active intervention and a 12 month follow-up period.

#### Completion rate

The long-term trial by Anderssen et al [75] reported a completion rate of 91% and 97% for the aerobic exercise and diet+aerobic exercise groups, respectively. Foster-Schubert et al [76] reported completion rates of 91% in the aerobic exercise group and 92% in the diet+aerobic exercise group. Four of the five trials that included follow-up assessments reported completion rates by intervention group [77], [80], [91], [92]. The median (range) completion rate for the aerobic exercise groups over the active interventions was 77% (44.7–89%) for the aerobic exercise groups and 67.8% (65.1–78%) for the diet+aerobic exercise groups. The median (range) completion rate at follow-up was 69% (40.4–82%) for aerobic exercise groups and 58.4% (51.2–80%) in the diet+aerobic exercise groups.

#### Diet

Long-term trials and trials with follow-up assessments generally employed energy restricted meal plan diets with behavioral counseling. Melanson et al [91] included 2 to 4 meal replacements/day, while Melanson et al [92]used 2 meals/day of a high fiber cereal in one intervention group.

#### Compliance with diet protocol

Anderssen et al [75] found greater reductions in energy intake assessed by food-frequency questionnaire in the diet+aerobic exercise group while Foster-Schubert et al [76] reported no change in energy intake assessed by food frequency questionnaire for either the aerobic exercise or diet+aerobic exercise groups. Four of the five trials with follow-up assessments reported dietary compliance. Trials by Melanson et al [91], [92] and Wing et al [80] showed greater reductions in energy intake assessed by food records in the diet+aerobic exercise groups compared with the aerobic exercise groups. Volpe et al [79] found no change in energy intake assessed by food records in either the aerobic exercise or diet+aerobic exercise groups.

#### Aerobic exercise mode/supervision

Modes employed in long-term trials comparing aerobic exercise with diet+aerobic exercise included brisk walking/jogging [75], and treadmill or cycle ergometer exercise [76]. Anderssen et al [75] provided supervised exercise while exercise was partially supervised in the trial by Foster-Schubert et al [76]. Aerobic exercise modes in trials with follow-up assessments included outdoor walking/jogging [77], [80], [91], [92] and the Nordic Track exercise machine [79]. Exercise was unsupervised in three trials [77], [91], [92],partially supervised in one trial [80] and supervised in one trial [79].

#### Aerobic exercise frequency

Exercise frequency in long-term trials was 3 days/wk. [75], [79] or 5 days/wk. [76]. Exercise frequency was 5 days/wk. in three trials [79], [91], [92], 3 days/wk. in one trial [80] and 3–5 days/wk. in one trial [77] that included follow-up assessments.

#### Aerobic exercise intensity

Anderssen et al [75] prescribed exercise intensity at 60–80% of peak heart rate while Foster-Schubert et al [76] prescribed 75%–85% of maximal VO_2_. Both trials by Melanson et al [91], [92] prescribed walking at a “moderate” pace while Skender et al [77] prescribed walking that was “vigorous but never strenuous”. Exercise intensity was not specified in two trials [79], [80].

#### Aerobic exercise duration

Anderssen et al [75] prescribed 60 minutes/day while Foster-Schubert et al [76] prescribed 45 min/day. Two trials with follow-up assessments prescribed individualized exercise duration designed to expend ∼500 kcal/session by completion of the intervention [91], [92]. Volpe et al [79] prescribed 30 minutes/day, Skender et al [77] prescribed 45 min/day, and Wing et al [80] prescribed walking at 3 miles/day.

#### Compliance with exercise protocol

Anderssen et al [75] reported that participants in the aerobic exercise group and diet+aerobic exercise groups attended 57% and 63% of scheduled exercise sessions, respectively. Foster-Schubert et al [76] found that participants in the aerobic exercise and diet+aerobic exercise groups achieved 80% and 85% of the weekly exercise goal (225 min/wk), respectively. Only one trial with follow-up assessments included information on exercise compliance [80]. Wing et al [80] reported that 75% of participants met the exercise goal by the end of the active intervention.

#### Intervention during follow-up

Both trials by Melanson et al [91], [92] asked participants to continue with the intervention assigned at baseline. Volpe et al [79] provided a Nordic Track exercise machine for home use but provided minimal investigator contact. Wing et al [80] provided continued behavioral sessions at a reduced frequency as compared with the active weight loss intervention

#### Outcomes assessed

In addition to body weight, four trials with comparisons of aerobic exercise and diet+aerobic exercise assessed waist circumference [75], [76], [79], [91],and three trials assessed body composition, one by underwater weight [79], one by DXA [76] and one by air displacement [91]. Four trials assessed blood lipids (total, HDL, LDL cholesterol and triglycerides [75], [79], [80], [91], 3 of which also assessed blood pressure [75], [79], [80] and one trial also assessed glucose, insulin and HbA_1c_ [80].

### Participant characteristics – Aerobic Exercise vs. Diet+Aerobic Exercise

#### Age

In the long-term trial by Anderssen et al [75] the mean age of the total sample was 40 yrs., while the age range in the trial by Skender et al [77], which provided both long-term and follow-up data was 25–45 yrs. Foster-Schubert et al [76] reported a mean age of 58.1 yrs. and 58.0 yrs., in the aerobic exercise and diet+aerobic exercise groups, respectively. Three of the five trials with follow up assessments reported mean age by intervention group [79], [80], [92]. The median (range) age was 43.5 yrs. (42.6–46.4 yrs.) in the aerobic exercise groups and 45.0 yrs. (42.1–46.3 yrs.) in the diet+aerobic exercise groups. Melanson et al 2004 [91] reported a mean age of the complete sample of 42.6 years.

#### Sex

The long-term trials by Foster-Schubert et al [76] and Anderssen et al [75] included 100% and 9.6% women, respectively. The median (range) percent women was 79% (47–88%) in the five trials that included follow-up assessments [77], [79], [80], [91], [92]. Only Volpe et al [79] reported separate results for men and women.

#### BMI

The mean BMI in the long-term trial by Anderssen et al [75] was 28.6 kg/m^2^ in both the aerobic exercise and diet+aerobic exercise groups. Foster-Schubert et al [76] reported a mean BMI of 30.7 kg/m^2^ in the aerobic exercise group and 31.0 kg/m^2^ in the diet+aerobic exercise group. Four of the 5 trials with follow-up assessments provided BMI by intervention group [79], [80], [91], [92]. The median (range) BMI was 30.8 kg/m^2^ (30.5–36.0 kg/m^2^) in aerobic exercise groups and 31.3 kg/m^2^ (30.5–35.7 kg/m^2^) in diet+aerobic exercise groups.

#### Minority representation

The minority representation in the one long-term trial reporting this information was 15% [76]. The minority representation in the one trial with follow-up assessments that presented this information was 24.9% [92].

#### Physical activity level

Both long-term trials [75], [76] and the 4 of 5 trials with follow-up assessments recruited sedentary participants [77], [79], [91], [92].

#### Health status

One long-term trial recruited healthy participants [76] while the other recruited participants with high BMI, and elevated diastolic blood pressure, total cholesterol and triglycerides [75]. All trials with follow-up assessments recruited healthy participants [77], [79], [80], [91], [92]; however, Wing et al [80] recruited participants with 1 or 2 biological parents with DM2.

## Results – Aerobic Exercise vs. Diet+Aerobic Exercise

Results for all trials with an aerobic exercise vs. diet+aerobic exercise comparison are presented in Table 5.

**Table 5:** Comparison of change in weight, body composition, and chronic disease risk factors in response to aerobic exercise compared with diet plus aerobic exercise.

Study	Variable	Baseline -end of active intervention		Baseline-end follow-up		During follow-up	
		AE	D+AE	AE	D+AE	AE	D+AE
**Long-term trials**							
Anderssen et al. (75)	WT (kg/%)	−0.9/−1.0	−5.6/−6.2^nr^	na	na	na	na
	WC (cm)	−1.9	−5.7 ^nr^	na	na	na	na
	TC (mmol/l)	−0.2	−0.48 ^nr^	na	na	na	na
	LDL-C (mmol/l)	−0.13	−0.39 ^nr^	na	na	na	na
	HDL-C (mmol/l)	0.04	+0.13 ^nr^	na	na	na	na
	TG (mmol/l)	−0.24	−0.58 ^nr^	na	na	na	na
	SBP (mmHg)	−2.2	−5.9 ^nr^	na	na	na	na
	DBP (mmHg)	−2.7	−5.2 ^nr^	na	na	na	na
Foster-Schubert et al. (76)	WT (kg/%)	−2/−2.4	−8.9/−10.8 *	na	na	na	na
	WC (cm)	−2	−7.5 *	na	na	na	na
	Fat mass (kg)	−2.1	−8.2 *	na	na	na	na
	Fat-free mass (kg)	+0.3	−0.4 *	na	na	na	na
**Follow-up trials**							
Melanson et al. 2004 (91)	WT (kg/%)	−0.1/−0.1	−6.9/−7.8 *	−0.4/−0.5	−7.1/−7.9 *	−0.3/−0.4	−0.20/−0.01 ^nr^
	WC (cm)	−1	−5.3 *	−1.8	−5.1 *	−0.8	+0.20 ^nr^
	Fat mass (kg)	−0.6	−6.3 *	−1.7	−7.6 *	−1.1	−1.3 ^nr^
	Fat-free mass (kg)	+0.7	−0.5 ^ns^	+1.5	+0.50 ^ns^	+0.8	+1.0 ^nr^
	TC (mmol/l)	−0.04	−0.96 ^nr^	−0.13	−0.34 ^nr^	−0.09	+0.62 ^nr^
	LDL-C (mmol/l)	−0.02	−0.22 ^nr^	−0.07	−0.17 ^nr^	−0.05	+0.05 ^nr^
	HDL-C (mmol/l)	−0.07	−0.05 ^nr^	−0.14	+0.008 ^nr^	−0.07	+0.058 ^nr^
	TG (mmol/l)	0.38	−0.34 ^nr^	−0.22	−0.43 ^nr^	−0.60	−0.90 ^nr^
Melanson et al. 2006 (92)	WT (kg/%)	−1.6/1.9%	−4.7/−5.4 (no cereal) *	−1.75/2	−5.7/−6.6 (no cereal) *	−0.15/−0.1	−1.0/−1.2 ^nr^
			−5.0/−6.0 (cereal) *		−6.2/−6.2% (cereal) *		−1.2/0.2 ^nr^
Skender et al. (77)	WT (kg/%)	−2.9/−3.1	−8.9/−8.9 ^ns^	−2.7/−2.9	−2.2/−2.2 ^ns^	0.2/0.2	6.7/6.7
Volpe et al. (79)	WT (kg)	Figures only					
Men	WC (cm)	−1.2	−4.7 ^ns^	−1.2	−1.9 ^ns^	−0.1	+2.8 ^nr^
	Fat mass (kg)	−1.6	−5.8 *	0.4	+2.0 ^ns^	−2.8	+3.0 ^nr^
	Fat-free mass (kg)	+0.3	−0.3 ^ns^	+0.20	+0.1 ^ns^	−0.1	+0.4 ^nr^
	TC (mmol/l)	−0.16	−0.27 ^ns^	−0.18	−0.04 ^ns^	−0.02	+0.23 ^nr^
	LDL-C (mmol/l)	+0.08	+0.02 ^ns^	+0.37	+0.15 ^ns^	+0.29	+0.13 ^nr^
	HDL-C (mmol/l)	−0.24	−0.22 ^ns^	−0.18	−0.13 ^ns^	+0.06	+0.90 ^nr^
	TG (mmol/l)	−0.32	−0.24 ^ns^	+0.07	−0.20 ^ns^	+0.39	−0.04 ^nr^
							
	SBP (mmHg)	−15.3	−11.8 ^ns^	−14.4	−15.6 ^ns^	+0.90	−4.8 ^nr^
	DBP (mmHg)	−7.7	−9.7 ^ns^	−7.9	−12.3^ns^	−0.20	−2.6 ^nr^
Women	WT (kg)	Figures only					
	WC (cm)	−3.6	+0.10 ^ns^	−3.9	−2.1 ^ns^	−0.30	−2.2 ^nr^
	Fat mass (kg)	−0.5	−4.2 *	−1	−1.9 *	−0.5	+2.3 ^nr^
	Fat-free mass (kg)	+0.1	+1.5 ^ns^	+0.9	+1.1 ^ns^	+0.80	−0.40 ^nr^
	TC (mmol/l)	+0.06	−0.07 ^ns^	+0.03	−0.18 ^ns^	−0.03	−0.11 ^nr^
	LDL-C (mmol/l)	+0.22	+0.38 ^ns^	+0.15	+0.10 ^ns^	−0.07	−0.28 ^nr^
	HDL-C (mmol/l)	−0.21	−0.26 ^ns^	−0.13	−0.23 ^ns^	+0.80	+0.03 ^nr^
	TG (mmol/l)	−0.06	−0.34 ^ns^	+0.03	−0.16 ^ns^	+0.09	+0.18 ^nr^
	SBP (mmHg)	−0.9	−3.1 ^ns^	+3.0	+2.1 ^ns^	+3.90	+5.20 ^nr^
	DBP (mmHg)	0.6	−4.30^ns^	+6.3	+1.2 ^ns^	+5.7	+5.5 ^nr^
Wing et al. (80)	WT (kg/%)	−2.1/−2.1	−10.3/−10.4 ^nr^	1/1	−2.5/−2.5 ^nr^	3.1/3.1	+7.8/+7.9 ^nr^
	TC (mmol/l)	+0.12	−0.33 ^nr^	+0.33	+0.09 ^nr^	+0.21	+0.42 ^nr^
	LDL-C (mmol/l)	+0.03	−0.13 ^nr^	+0.22	+0.12 ^nr^	+0.19	+0.25 ^nr^
	HDL−C (mmol/l)	+0.02	+0.02 ^nr^	+0.05	+0.02 ^nr^	+0.03	0.00 ^nr^
	TG (mmol/l)	+0.12	−0.69 ^nr^	+0.33	−0.28 ^nr^	+0.21	+0.41 ^nr^
	Glucose (mmol/l)	0	−0.20 ^nr^	+0.40	+0.50 ^nr^	+0.40	+0.70 ^nr^
	Insulin (pmol/l)	−4	−37.0 ^nr^	+43.0	−12.0 ^nr^	+47.0	+0.25 ^nr^
	HbA_1c_ (%)	+0.1	+0.03 ^nr^	−0.10	+0.04 ^nr^	−0.20	+0.01 ^nr^
	SBP (mmHg)	−2.4	−12.3 ^nr^	+0.9	−4.8 ^nr^	+3.30	+7.5 ^nr^
	DBP (mmHg)	−1.7	−6.9 ^nr^	+2.0	−0.2 ^nr^	+3.7	+6.7 ^nr^

AE  =  aerobic exercise, D+AE  =  diet plus aerobic exercise, WT  =  body weight, WC  =  waist circumference, TC  =  total cholesterol, LDL  =  low density lipoprotein cholesterol, HDL  =  high density lipoprotein cholesterol, TG  =  triglycerides, na  =  not assessed, ns  =  between group difference for change non- significant, nr  =  between group difference for change not reported.

*between group difference for change significant at p <0.05 or greater.

### Long-term trials

#### Body weight

Anderssen et al [75] reported decreased body weight in both the aerobic exercise (−1.0%) and diet+aerobic exercise groups (−6.2%); however the statistical significance of the between group difference was not reported. Foster-Schubert et al [76] reported significantly greater weight loss in the diet+aerobic exercise (−10.8%) compared with the aerobic exercise group (−2.4%).

#### Waist circumference

Anderssen et al [75] reported decreased waist circumference in both the aerobic exercise (−1.9 cm) and diet+aerobic exercise groups (−5.7 cm); however statistical significance of the between group difference was not reported. Foster-Schubert et al [76] reported significantly greater reductions in waist circumference in the diet+aerobic exercise (−7.0 cm) compared with the aerobic exercise group (−2.1 cm).

#### Fat/fat-free mass

Foster-Schubert et al [76] reported significant between group differences for both change in fat mass (aerobic exercise = −2.1 kg, diet+aerobic exercise = −8.2 kg) and fat-free mass (aerobic exercise = +0.3 kg, diet+aerobic exercise = −0.4 kg).

#### Chronic disease risk factors

Anderssen et al [75] reported decreases in both systolic (aerobic exercise  = −2.2 mmHg; diet+aerobic exercise = −5.9 mmHg) and diastolic blood pressure (aerobic exercise  = −2.7 mmHg; diet+aerobic exercise  = −5.2 mmHg) in diet and diet+aerobic exercise groups. Larger decreases in total cholesterol (aerobic exercise  = −0.20 mmol/l, diet+aerobic exercise  = −0.48 mmol), LDL cholesterol (aerobic exercise  = −0.13 mmol/l; diet+aerobic exercise  = −0.39 mmol/l) and trigylcerides (aerobic exercise  = −0.24 mmol/l; diet+aerobic exercise  = −0.58 mmol/l) were observed in diet+aerobic exercise groups compared to aerobic exercise alone. The increase in HDL cholesterol was greater in the diet+aerobic exercise (+0.13 mmol/l) compared with aerobic exercise only (+0.04 mmol/l). Anderssen et al [75] did not report the statistical significance of the between group differences.

### Trials with follow-up assessments

#### Body weight

Four of the 5 trials that assessed weight change, both after an active intervention and a follow-up period, reported or provided numerical data with which percent weight change could be calculated [77], [80], [91], [92]. The median (range) for weight loss from baseline to the completion of the weight loss intervention was 2% (0.1–3%) in aerobic exercise groups and 7.8% (3.2–24.6%) in diet+aerobic exercise groups. Weight loss in the diet+aerobic exercise groups was significantly greater than aerobic exercise groups in the 3 trials providing this information [77], [91], [92]. No trials reported weight loss ≥5% in the aerobic exercise group while weight loss ≥5% was observed in all diet+aerobic exercise groups. Volpe et al [79] presented weight loss data only in figures. In women, weight loss was significantly greater in the diet+aerobic exercise compared with the aerobic exercise group; however, no significant between group differences in weight loss were observed for men.

The median (range) percent weight loss from baseline to the completion of follow-up was 1.5% (0.5–2.9%) in aerobic exercise groups and 5.7% (2.2–7.1%) in diet+aerobic exercise groups in the 4 trials reporting this information [77], [80], [91], [92]. Weight loss was significantly greater in the diet+aerobic exercise compared with aerobic exercise only groups in 2 [91], [92] of 4 trials providing this data [77], [79], [91], [92]. Volpe et al [79] found no significant between group differences for weight loss from baseline to the completion of follow-up in men or women. It is interesting to note that the 2 trials reporting significantly greater weight loss from baseline to the end of follow-up in the diet+aerobic exercise compared with aerobic exercise group included only a 12 week follow-up after 12 weeks of weight loss [91], [92].

Four trials provided numerical data on weight change during follow-up [77], [80], [91], [92]. Two trials reported by Melanson et al [91], [92] with relatively short duration of follow-up (12 wks.) reported small continued weight loss (∼1 kg) during the follow-up period in both aerobic exercise and diet+aerobic exercise groups. Skender et al [77] reported the percent of lost weight regained at follow-up was 7% in the aerobic exercise group and 75% in the diet+aerobic exercise group. In the trial by Wing et al [80] the percentage of lost weight regained at follow-up was 55% and 76% for the aerobic exercise and diet+aerobic exercise groups, respectively.

#### Waist circumference

Two trials reported change in waist circumference [79], [91]. Melanson et al [91] reported significantly greater reductions in waist circumference from baseline to the completion of weight loss intervention (12 wks.) in the diet+aerobic exercise (−5.3 cm) compared with the aerobic exercise group (−1.0 cm) which were maintained or slightly enhanced at a 24 wk. follow-up (diet+aerobic exercise  = −5.1 cm; aerobic exercise  = −1.8 cm). Volpe et al [79] found no significant between group difference for change in waist circumference in either men (aerobic exercise  = −1.2 cm, diet+aerobic exercise  = −4.7 cm) or women (aerobic exercise  = −3.6 cm, diet+aerobic exercise  = +0.1 cm) at the end of the active intervention (6 mos.) or follow-up at 12 mos. (men: aerobic exercise  = −1.2 cm, diet+aerobic exercise  = −1.9 cm, (women: aerobic exercise  = −3.9 cm, diet+aerobic exercise  = −2.1 cm).

#### Fat mass/fat-free mass

Two trials reported change in fat and fat-free mass [79], [91]. Melanson et al [91] reported significantly greater reductions in fat mass in the diet+aerobic exercise (−6.3 kg) compared with the aerobic exercise group (−0.6 kg) from baseline to the end of a 12 wk. weight loss intervention which were maintained or slightly enhanced at a 24 wk. follow-up (diet+aerobic exercise = −7.6 kg; aerobic exercise  = −1.7 kg). Volpe et al [79] reported significantly greater decreases in fat mass in the diet+aerobic exercise compared with the aerobic exercise group for both men (aerobic exercise  = −1.6 kg, diet+aerobic exercise  = −5.8 kg) and women (aerobic exercise  = −0.5 kg, diet+aerobic exercise  = −4.2 kg) at the completion of the 6 month intervention. However, these differences were not shown at the 12 mo. follow-up in either men (aerobic exercise  = +0.4 kg, diet+aerobic exercise  = −2.8 kg) or women (aerobic exercise  = −1.0 kg, diet+aerobic exercise  = −1.9 kg).

Melanson et al [91] observed no significant changes in fat-free mass over the 12 week active intervention in either the aerobic exercise or diet+aerobic exercise groups. However, at follow-up, fat-free mass was significantly increased in the aerobic exercise group (+1.5 kg) but not in the diet+aerobic exercise group (+0.5 kg), with no significant between group differences. Volpe et al [79] observed small non-significant increases in fat-free on completion of the active intervention and at follow up in both men and women, with no significant between group differences.

#### Chronic Disease Risk Factors

Three trials reported chronic disease risk factors [79], [80], [91]. At completion of the active intervention (6 months), Wing et al [80] reported no significant within group changes in the aerobic exercise group in total, HDL and LDL cholesterol, triglycerides, systolic and diastolic blood pressure, fasting insulin, fasting glucose or HbA_1C._ At the 24 month follow-up significant increases in total cholesterol, glucose and insulin were observed, with no significant changes in any of the other risk factors. In the diet+aerobic exercise group there were significant reductions in total cholesterol, triglycerides, systolic blood and diastolic blood pressure, glucose, and insulin at 6 months. However, at follow-up (24 months) no significant changes were observed in any of these parameters and HbA_1C_, and fasting glucose were both significantly increased over baseline. Thus, risk factor changes associated with the active intervention were not maintained at follow-up. The statistical significance of between group for change at the end of the active intervention or follow-up were not reported [80]. Volpe et al [79] reported no significant changes in systolic blood or diastolic blood pressure, total, HDL or LDL cholesterol, or triglycerides in either the aerobic exercise or diet+aerobic exercise groups after completion of the active intervention (6 months) or at follow-up (12 months) in either men or women. Melanson et al [91] did not report the statistical significance for between group changes; however, reductions in total and LDL cholesterol observed during the active intervention were maintained or enhanced in the aerobic exercise but not in the diet+aerobic exercise group.

### Additional diet/diet+exercise comparisons

Trials by Hunter et al [84], Wadden et al [89] and Svendsen et al [93] provided comparisons of various combinations of diet, aerobic and resistance exercise (Table 6).

**Table 6:** Comparison of change in weight, body composition, and chronic disease risk factors in response to combinations of diet, aerobic and resistance exercise.

Study		Variable	Baseline - end of active intervention		Baseline - end Follow-up		During follow-up	
			**D**	**D+RE**	**D**	**D+RE**	**D**	**D+RE**
Hunter et al. (84)		WT (kg/%)	na	na	na	na	+6.4/9.8	Adhere = +3.9/5.9 *
		WT(kg/%)	na	na	na	na	+6.4/9.8	Non-Adhere = +6.2/9.5 ^ns^
		WC (cm)	na	na	na	na	+5.5	Adhere = +2.9 ^ns^
		WC (cm)	na	na	na	na	+5.5	Non-Adhere = +5.3 ^ns^
		% Fat	na	na	na	na	+5.3	Adhere = +4.4 *
		% Fat	na	na	na	na	+5.3	Non-Adhere = +5.8 ^ns^
		Visceral fat (cm^2^)	na	na	na	na	+12.4 **	Adhere = −0.4 ^ns^ within
		Visceral fat (cm^2^)	na	na	na	na	+12.4 **	Non-Adhere = +10.4 ^ns^ within
Wadden et al. (89)		WT (kg/%)	−15.3/−16.3	−17.3/−17.3 ^ns^	−6.9/−7.7	−10.1/−10.0 ^ns^	+8.4/8.6	+7.2/7.3 ^nr^
							
			**D+AE**	**D+RE**	**D+AE**	**D+RE**	**D+AE**	**D+RE**
Hunter et al. (84)		WT (kg/%)	na	na	na	na	Adhere = +3.1/4.9%	Adhere = +3.9/5.9 ^ns^
		WT(kg/%)	na	na	na	na	Non-Adhere = +7.1/10.9	Non-Adhere = +6.2/9.5 ^ns^
		WC (cm)	na	na	na	na	Adhere = +1.9	Adhere = +2.9 ^ns^
		WC (cm)	na	na	na	na	Non-Adhere = +7.9	Non-Adhere = +5.3 ^ns^
		% Fat	na	na	na	na	Adhere = +3.4	Adhere = +4.4 *
		% Fat	na	na	na	na	Non-Adhere = +6.4	Non-Adhere = +5.8 ^ns^
		Visceral fat (cm^2^)	na	na	na	na	Adhere = +0.8 ^ns^ within	Adhere = −0.4 ^ns^ within
		Visceral fat (cm^2^)	na	na	na	na	Non-Adhere = +25.5 **	Non-Adhere = +10.4 ^ns^ within
Wadden et al. (89)		WT (kg/%)	−13.5/−13.9	−17.3/−17.3 ^ns^	−8.5/−8.6	−10.1/−10.0 ^ns^	+5/5.3	+7.2/7.3 ^nr^
			**D**	**D+AE+RE**	**D**	**D+AE+RE**	**D**	**D+AE+RE**
Wadden et al. (89)		WT (kg/%)	−15.3/−16.3	−16.6/−17.6 ^ns^	−6.9/−7.7	+8.6/8.6 ^ns^	+8.4/8.6	+8.0/9.0 ^nr^
			**D+AE**	**D+AE+RE**	**D+AE**	**D+AE+RE**	**D+AE**	**D+AE+RE**
Wadden et al. (89)		WT (kg/%)	−13.5/13.9	−16.6/−17.6 ^ns^	−8.5/8.6	+8.6/8.6 ^ns^	+5/5.3	+8.0/9.0 ^nr^
			**D+RE**	**D+AE+RE**	**D+RE**	**D+AE+RE**	**D+RE**	**D+AE+RE**
Wadden et al. (89)		WT (kg/%)	−17.3/−17.3	−16.6/−17.6 ^ns^	−10.1/−10.0	+8.6/8.6 ^ns^	+7.2/7.3	+8.0/9.0 ^nr^

D =  diet, D+AE  =  diet plus aerobic exercise, D+RE  =  diet plus resistance exercise, D+AE+RE  =  diet plus aerobic plus resistance exercise, WT  =  body weight, WC  =  waist circumference, na  =  not assessed, ns  =  between group difference for change non- significant, nr  =  between group difference for change not reported.

*between group difference for change significant at p<0.05 or greater.

**within group change significant at p<0.05 or greater.

Hunter et al [84] reassessed weight, waist circumference, and body composition (DXA, computed tomography) in a sample of initially overweight and obese women one-year following completion of a weight loss intervention by diet, diet+resistance exercise or diet+aerobic exercise. The basic study design and descriptions and results of the diet and aerobic training components of this trial were presented previously. Here we present results for diet vs. diet+resistance training and diet+aerobic vs. diet+resistance training. Supervised resistance exercise was ramped to 2 sets of 10 exercises at 80% of one repetition maximum, 3 days/week by week four. During the weight loss phase compliance with the exercise protocol was required to remain in the study. The weight loss intervention was continued until participants reached a BMI of 25 kg/m^2^ (mean 154 days) at which time they were asked to continue with their original group assignment with supervised exercise frequency reduced to two days/week for exercise groups over the next 12 months. All groups were given instruction on following a balanced diet that focused on consumption of low-density foods. Exercise during the follow-up period was not required, thus two groups were formed; participants who adhered (≥60% of scheduled sessions) and those who did not adhere (<60% of scheduled sessions) to the exercise protocols. Results indicated that body weight increased significantly during the one-year follow-up, with a significant group by time interaction. No significant between group differences for change in any outcomes were observed in participants who adhered to either the aerobic or resistance exercise protocols. However, participants who adhered to the aerobic (+4.9%) or resistance exercise (+5.9%) gained significantly less weight than any of the other 3 groups (aerobic non-adhere  = +10.9%; resistance non-adhere  = +9.5%; no exericise  = 9.8%). Similar results were observed for change in % fat in that participants who adhered to either aerobic or resistance exercise showed smaller increases in % fat than the other 3 groups. Waist circumference increased in all groups with no significant between group differences. In addition, the change in visceral fat in groups who were adherent to exercise was non-significant (aerobic  = +1.6%; resistance = −0.9%) while significant increases in visceral fat were observed in the two non-adherence groups (aerobic  = +54.4%; resistance  = +29.4%) and the no exercise group (+24.8%). Thus, irrespective of the mode of weight loss, increases in weight, % fat and visceral fat were minimized only in those who continued with either aerobic or resistance exercise.

Wadden et al [89] reported results for weight change from a 1-year follow-up in a sample of overweight/obese women (age ∼41 yrs; BMI ∼36 kg/m^2^) who completed a weight loss intervention [89] employing combinations of diet, aerobic and resistance exercise. Participants were randomized to 4 conditions (diet: n = 29, diet+aerobic exercise: n = 31, diet+resistance exercise: n = 31 and diet + aerobic+resistance exercise: n = 29) for a 48 week weight loss phase and followed-up at 1-year during which time there was no intervention. Diet was a 16 week VLCD (∼925 kcal/day) followed by a conventional energy restricted diet (1,200 to 1,500 kcal/day) for 31 weeks with behavioral counseling; weekly for 28 weeks, bi-weekly for 20 weeks. Participants in aerobic exercise groups were prescribed 3 supervised sessions of step aerobics/wk. over the first 28 weeks and 2 supervised sessions/wk. over weeks 29–48. Participants in resistance exercise groups were prescribed 3 supervised sessions of 10 exercises that progressed in resistance when more than 14 repetitions could be performed on two consecutive sets. Weight loss over the first 48 weeks was significant in all intervention groups (diet  = −16.3%, diet + aerobic exercise  = −13.9%, diet+resistance exercise  = −17.3%, diet+aerobic+resistance exercise  = −17.6%) with no significant between group differences. Weight loss at 1-year follow-up was also significant in all intervention groups (diet  = −7.7%, diet + aerobic exercise  = −8.6%, diet+resistance exercise  = −10.0%, diet+aerobic+resistance exercise  = −8.6%) with no significant between group differences. All intervention groups regained similar amounts of weight following weight loss (diet  = +8.4 kg, 54.9% of lost weight, diet + aerobic exercise  = +5.0 kg, 37% of lost weight, diet+resistance exercise  = +7.2 kg, 41.6% of lost weight, diet+aerobic+resistance exercise  = +8 kg, 48.2% of lost weight). Weight loss in 27 participants who reported exercising regularly in the 4 months preceding the one year follow-up (12.1 kg) was significantly greater than 22 participants who reported no regular exercise (6.1 kg). Weight regain over the follow-up period was also significantly less in exercisers (5.5 kg) compared with non-exercisers (8.4 kg).

Svendsen et al. [93] randomly assigned overweight and obese post-menopausal women (mean age  = ∼54 yrs.) to one of three groups: diet, diet+aerobic+resistance exercise, or a no intervention control: n = 21). Participants were assessed 24 weeks following completion of a 12 week weight loss intervention. During the weight loss phase energy intake was restricted to 4.2 MJ/d (1.6 MJ/d formula diet +2.6 MJ/d freely chosen food). Exercise was supervised and included 3–60 to 90 minute session per week of combined aerobic (cycle ergometer at ≥70% VO_2_ max) and resistance exercise at ≥65% maximal lifting capacity). During the 24-week follow-up, participants did not participate in any intervention but were encouraged to continue exercise and monitor energy intake to maintain weight loss. Main outcome data were presented in figures only. Both the diet and diet+aerobic+resistance exercise groups showed significant reductions in weight and fat mass at both 12 and 24 weeks (∼8 kg) compared with control with little or no regain in weight or fat mass in either group over the follow-up period. However, fat-free mass was significantly reduced in the diet only group compared with diet+aerobic+resistance exercise and control groups at both 12 and 24 weeks. Participant in the diet+aerobic+resistance exercise group that were self-reported participating in exercise during the follow-up period based on self-report showed significantly greater reductions in weight (exerciser  = 10.9 kg; non-exerciser  = 6.6 kg) and fat mass (exerciser  = 10.0 kg, non-exerciser  = 5.4 kg) compared with non-exercisers in this group. Total and LDL cholesterol decreased significantly during weight loss in both the diet and diet+aerobic+resistance exercise groups compared with controls; however, these changes were not maintained at the 24 week follow-up. Triglycerides decreased significantly during weight loss in both active intervention groups compared with control. Unlike total and LDL cholesterol, the change in triglycerides was maintained at 24 weeks. HDL cholesterol was unchanged in any intervention group at 12 weeks. At 24 weeks HDL cholesterol was significantly higher in both active intervention groups compared with control.

#### Risk of bias

The risk of bias is presented in Table 7. The description of the procedures for random sequence generation were unclear in 15 of 20 (75%) of trials included in this review [5], [75], [77]–[80], [82]–[86], [88]–[90], [93]. Three trials adequately described randomization procedures and were considered low risk of bias [76], [91], [92]. Randomization bias was high in two trials; one trail allowed participants who refused their random assignment to select their intervention group [81], and one trial employed a “pseudo randomized” process [87]. No trials described procedures for allocation concealment. The blinding of participants and personnel is not feasible in a diet/diet+ exercise trial. While blinding of personnel performing outcome assessments is feasible, these procedures were employed in only one trial [76]. The risk of attrition bias was high in five trials where the study sample represented approximately 60% or less (range 48–61%) of those randomized at baseline [77], [81], [86], [89], [91]. The most important other source of potential bias was inadequate statistical power. Only 3 of 20 trials (15%) reported adequate statistical power to detect between group differences for change in body weight [76], [79], [83]; however, one trial did not specifically indicate that the reported power was based on the body weight outcome [79]. Nine of the 17 trials (∼53%) that did not report statistical power were conducted in small samples (∼20 participants/group or less) and thus likely to be inadequately powered to detect between group differences [5], [77], [78], [84], [86]–[88], [90], [91].

**Table 7:** Study Risk of bias.

Study	Random Sequence Generation (selection bias)	Allocation concealment (selection bias)	Blinding participants and personnel (performance bias)	Blinding of outcome assessment (detection bias)	Incomplete outcome data (attrition bias)	Selective reporting (reporting bias)	Other bias
Anderssen et al. (75)	Unclear	NR	NA	NR	Low risk	Low risk	Unclear
Andreou et al. (83)	Unclear	NR	NA	NR	Low risk	Low risk	Low risk
Dengel et al. (81)	High risk	NR	NA	NR	High risk	Low risk	Unclear
Foster-Schubert et al. (76)	Low risk	NR	NA	Low risk	Low risk	Low risk	Low risk
Hunter et al. (84)	Unclear	NR	NA	NR	Low risk	Low risk	High risk
Melanson et al. 2004 (91)	Low risk	NR	NA	NR	High risk	Low risk	High risk
Melanson et al. 2006 (92)	Low risk	NR	NA	NR	Low risk	Low risk	Unclear
Molenar et al. (85)	Unclear	NR	NA	NR	Low risk	Low risk	Low risk
Neumark-Sztainer et al. (86)	Unclear	NR	NA	NR	High risk	Low risk	High risk
Pritchard et al. (78)	Unclear	NR	NA	NR	Low risk	Low risk	High risk
Racette et al. (5)	Unclear	NR	NA	NR	Low risk	Low risk	High risk
Snel et al. (87)	High risk	NR	NA	NR	Low risk	Low risk	High risk
Skender et al. (77)	Unclear	NR	NA	NR	High risk	Low risk	High risk
Svendsen et al. (93)	Unclear	NR	NA	NR	Low risk	Low risk	Unclear
Van Aggel-Leijssen et al. (88)	Unclear	NR	NA	NR	Low risk	Low risk	High risk
Vople et al. (79)	Unclear	NR	NA	NR	Unclear	Low risk	Low risk
Wadden et al. (89)	Unclear	NR	NA	NR	High risk	Low risk	Unclear
Wang et al. (90)	Unclear	NR	NA	NR	Low risk	Low risk	High risk
Wing et al. (80)	Unclear	NR	NA	NR	Low risk	Low risk	Unclear
Wood et al. (82)	Unclear	NR	NA	NR	Low risk	Low risk	Unclear

NR  =  not reported, NA  =  not applicable.

## Discussion

### Summary of evidence

In this paper we systematically reviewed 20 randomized trials to address the question: Does the method of weight loss, i.e., energy restriction, exercise (aerobic or resistance), and the various combinations effect long-term changes in weight, body composition or chronic disease risk factors in overweight or obese adults?

#### Diet vs. Aerobic Exercise

Long-term weight loss tended to be greater with diet compared with aerobic exercise with ad libitum eating. This observation is in agreement with results from other reviews which have shown only modest weight loss with aerobic exercise [69], [70], [94]–[96]. However, most trials of aerobic exercise for weight loss are limited in that the energy expenditure of exercise is neither rigorously controlled or accurately measured, and the level of prescribed exercise is insufficient to produce significant weight loss [97]. With the exception of the trial by Racette et al. [5], long-term trials included in this review prescribed insufficient levels of exercise and observed weight loss of ∼3% or less in exercise only groups. In contrast, Racette et al. [5] prescribed exercise energy expenditure (∼1,900 kcal/wk) to match the energy deficit induced by diet and observed clinically significant weight loss in the aerobic exercise group (−8.4%) that was not significantly different from weight loss in the diet only group (−10.4%). The results of Racette et al [5] are in agreement with those from short-term trials (12-14 wks) which have shown clinically significant weight loss (6.0–7.7%) with equivalent energy deficits induced by either diet or aerobic exercise of 500–700 kcal/day [4], [98] and longer term trials (10–16 months) which have shown weight loss induced by exercise alone (≥2000 kcal/wk) ranging from 4.3–5.7% [6], [7]. The results of the long-term trial of Racette et al [5], and from other short and long-term trials in the literature [4], [6], [7], [98], suggest that clinically significant weight loss can be achieved with aerobic exercise alone when exercise energy deficits are similar in magnitude to those induced by energy restriction.

Similar to changes in body weight, reductions in both fat mass and waist circumference in long-term trials tended to be greater in the diet only compared with the exercise only groups. Fat-free mass in long-term trials tended to be preserved in exercise groups, which may be a function of the minimal levels of weight loss observed in the aerobic exercise groups [5], [76]. However, in our recently completed 10 month aerobic exercise trial (5 days/wk., 400 or 600 kcal/day) we showed fat-free mass was unchanged in the 400 kcal/day group and increased slightly (1.1%) in the 600 kcal/day group despite weight loss of 4.3% and 5.7% in the 400 and 600 kcal/day groups, respectively [6]. In this review, we identified only 1 long-term trial that compared changes in chronic disease risk factors between diet and aerobic exercise. Although statistical significance of between group changes was not reported, there were no obvious between group differences for changes in risk factors including total, LDL and HDL cholesterol, triglycerides and blood pressure [75].

Results from trials investigating differences in weight regain following weight loss by diet or aerobic exercise were mixed [77], [79], [80]. Trials, with follow-up periods ranging from 6 to 18 months, reported less weight regain in the aerobic exercise compared with the diet group [77], less weight regain in the diet compared with the aerobic exercise group [80], and no between group differences in weight regain between diet and aerobic exercise groups [79]. Only one of these trials included an intervention during the follow-up period consisting of behavioral group meetings at a reduced frequency from the active intervention [80]. Results from these trials are difficult to interpret in terms of assessing the impact of differences in weight loss method (diet or aerobic exercise) on weight change during follow-up, as weight loss at the end of the active intervention was minimal in the aerobic exercise groups (∼2.6%) compared with weight loss in the diet only groups (∼8%). Thus, without clinically significant weight loss in the aerobic exercise groups, the impact of weight loss by this mode on subsequent weight regain, and or subsequent changes in body composition cannot be evaluated. Results from the 2 trials that evaluated the effect of diet or aerobic exercise interventions on chronic disease risk factors at follow-up were also mixed [79], [80]. One trial observed no significant changes in risk factors in either diet or aerobic exercise groups on completion of the active intervention or at follow-up [79], while one trial reported beneficial changes in risk factors on completion of the active intervention in the diet group which were not maintained at follow-up [80]. The limited available data suggest no sex differences in weight, body composition or chronic disease risk factors in response to diet or aerobic exercise interventions [79].

#### Diet vs. Diet+Aerobic exercise

Adding aerobic exercise to an energy restricted diet tended to result in slightly greater long-term weight loss than energy restricted diet alone. Although weight loss was significantly greater in the diet+aerobic exercise group in 2 of the 4 trials presenting this data, the additional weight loss achieved when adding aerobic exercise to an energy restricted diet was quite small, i.e., ∼2.2% [76], [82]. These findings are in general agreement with a previous meta-analysis by Wu et al [66] who reported greater long-term weight loss with interventions including diet+aerobic exercise compared with diet alone with small between group differences (pooled mean difference  = 1.14 kg). As previously discussed, the exercise prescribed and/or actually completed in these trials (i.e. ∼155 min/wk) was likely insufficient to elicit significant additional weight loss [75], [76], [81], [82]. Significantly greater long-term weight loss with diet+aerobic exercise compared to diet alone has been shown in both men and women [82].

Reductions in both fat mass and waist circumference were greater in diet+aerobic exercise compared with diet only groups; however, as observed with body weight, the between group differences in both fat mass and waist circumference, even when statistically significant, were small (waist circumference  = 2.0–3.0 cm, fat mass  = 0.1–2.7 kg) [76], [81], [82]. Reductions in fat-free mass were smaller in diet+aerobic exercise compared with the diet only groups; however, no significant between group differences were noted. However, other reviews [95], [96] and results from short-term [99]–[101] and longer duration trials [6] have suggested that aerobic exercise combined with an energy restricted diet preserves fat-free mass during weight loss. One trial reported greater reductions in fat mass with diet+aerobic exercise compared with in men, but not in women [82].

We identified only 2 long-term trials that compared changes in chronic disease risk factors between diet and diet+aerobic exercise [75], [82]. Although statistical significance of between group changes was not reported, Anderssen et al [75] showed beneficial changes in total, LDL, and HDL cholesterol, triglycerides, and blood pressure that were of greater magnitude in the diet+aerobic exercise compared with the diet only group. Wood et al [82] observed significantly greater increases in HDL-cholesterol and reductions in triglycerides in men and greater increases in HDL-cholesterol in women in the diet+aerobic exercise compared with diet only groups. Thus, we found limited evidence to suggest a difference for change in chronic disease risk factors when weight loss is achieved with diet alone or a combination of diet+aerobic exercise.

Results from trials investigating differences in weight regain following weight loss by diet or diet+aerobic exercise do not suggest an obvious benefit for weight loss by either weight loss method [77], [80], [83], [85], [87]–[90]. In agreement with the long-term trials described previously, weight loss was generally greater following completion of the active intervention in the diet+aerobic exercise (∼14%) compared with the diet only groups (∼12%). However, during the follow-up period, which ranged from 18 weeks to 12 months, the percentage of lost weight that was regained was similar in both the diet (∼58%) and diet+aerobic exercise groups (∼53%). Weight regain was similar in the diet and diet+aerobic exercise groups in the 2 trials that included an intervention during follow-up. One trial by [80] included behavioral group meetings at a reduced frequency from the active intervention and one trial included low intensity exercise (40% VO_2_ max); however compliance with exercise was low (57%) [88]. Wadden et al [89] did not prescribe exercise during follow-up; however, noted that weight regain was significantly reduced in participants who self-reported exercise during follow-up compared with those who did not, irrespective of group assignment (diet or diet+aerobic exercise). The finding of no difference in weight regain with weight loss by diet or diet+aerobic exercise is in agreement with those of several previous reviews [65]–[67], [70].

Results for the change in waist circumference between diet and diet+aerobic exercise were mixed. Greater reductions in waist circumference were observed in diet+aerobic exercise groups on completion of the active intervention in some [83], [87] but not all trials [79]. One trial with a short follow-up period (18 wks) showed continued decreases in waist circumference during follow-up in both diet and diet+aerobic exercise groups [83], while in 2 trials the reductions in waist circumference observed at the end of the active intervention were eliminated at the completion of the follow–up period [79], [87]. One trial reported a significantly greater decrease in waist circumference in a diet+aerobic exercise group compared with diet at completion of the active intervention in men, but not in women; however, the sex differences was not observed at follow-up [79]. Results for changes in fat mass or percent body fat between diet and diet+aerobic exercise were also mixed with some [79], [83], [87], but not all trials [88] reporting greater decreases in fat mass/percent fat with diet+aerobic exercise compared with diet alone. The decrease in fat mass in the diet+aerobic exercise groups was significantly greater than diet alone in women but not men at the end of the acive intervention, but not at follow-up in the one trial reporting this data [79]. Fat mass/percent fat increased during the follow-up period in both diet and diet+aerobic exercise with no between group differences in these trials with the exception of the one trial with a short follow-up period (18 wks.) where fat mass decreased slightly in both intervention groups [83]. There were no between group differences for change in fat-free mass either at the end of the active intervention or at follow-up in the 2 trials reporting this variable [79], [88]. We found no evidence for differences between diet and diet+aerobic exercise for changes in chronic disease risk factors or any sex differences at the end of an active intervention or at follow-up in the 3 trials presenting risk factor data [79], [80], [87]. One trial observed significant reductions in total cholesterol, triglycerides, blood pressure, glucose and insulin at the completion of the active intervention in the diet only group that were not maintained at follow-up [80].

#### Aerobic exercise vs. Diet+aerobic exercise

As discussed previously, trials which have included aerobic exercise groups have generally prescribed aerobic exercise that is insufficient to elicit significant weight loss. The 2 long-term trials comparing aerobic exercise with diet+aerobic exercise prescribed ∼185 min/wk and observed weight loss in the aerobic exercise groups of ≤2.4% over 12 months, which was considerably less than the weight loss observed in the diet+aerobic exercise groups (∼6% and ∼11%) over the same time frame [75], [76]. Likewise, reductions in waist circumference were also larger in diet+aerobic exercise compared aerobic exercise only groups [75], [76]. In the one trial that assessed changes in body composition, reductions in fat mass were significantly greater in the diet+aerobic exercise compared with the aerobic exercise only group while fat-free mass maintained in the aerobic exercise group and decreased in the diet+aerobic exercise groups [76]. The results of the one long-term trial that assessed chronic disease risk factors suggested greater reductions in total and LDL cholesterol, triglycerides, and blood pressure and greater increases in HDL cholesterol in the diet+aerobic exercise compared with the aerobic exercise group [75].

Results for trials investigating weight regain following weight loss by aerobic exercise or diet+aerobic exercise were mixed [77], [80], [91], [92]. Two trials with a relatively short active intervention (12 wks.) and follow-up period (12 wks.) both observed significantly greater weight loss in the diet+aerobic exercise compared with the aerobic exercise only group at the end of the active intervention and at follow-up [91], [92]. The two trials with longer duration follow-up periods (12 &18 mos.) reported no differences between aerobic exercise and diet+aerobic exercise groups for weight change at the end of the active intervention or at follow-up with both groups regaining most of their lost weight during follow-up [77], [80]. Only the trial by Wing et al [80] included an intervention during the follow-up period. Similar to changes in body weight, the reductions in waist circumference and fat mass were significantly greater in the diet+aerobic exercise compared with aerobic exercise at the end of the active intervention and at follow-up when the intervention (12 wks.) and follow-up periods (12 wks.) were relatively short [91]. However, the trials with longer duration interventions and follow-up found no between group difference for change in waist circumference at either time point [79]. Greater reductions in fat mass at the end of the active intervention were shown in both men and women in the diet+aerobic compared with the aerobic exercise group, that were maintained at follow-up in men but not in women [79]. No trials showed any between group differences for change in fat-free mass at the end of the active intervention or at follow-up [79], [91]. As previously discussed, weight loss (range 0.1% to 3.1%) as well as changes in body composition in the aerobic exercise groups in all these trials was minimal, making it impossible to evaluate the effect of weight loss by aerobic exercise on subsequent weight regain. Results from the 3 trials that compared change in risk factors between aerobic exercise and diet + aerobic exercise were mixed [79], [80], [91]. One trial with a relatively short active intervention (12 wks.) and follow-up period (12 wks.) observed significantly greater reductions in total and LDL-cholesterol and triglycerides but not in HDL-cholesterol in the diet+aerobic exercise compared with the aerobic exercise group both at the end of the active intervention and at follow-up[91]. However, trials with longer active intervention (6 mos.) and follow-up periods (6 or 18 mos.) observed both no change in lipid parameters or blood pressure at the end of the active intervention or at follow-up [79] or greater changes in these parameters at the end of the active intervention in the diet+aerobic exercise compared with aerobic exercise groups in both men and women, that were not maintained at follow-up.

#### Other comparisons

A limited number of trials have compared the effect of aerobic and resistance training, or combinations of diet with aerobic and resistance training on weight loss [84], [89], [93]. No advantage in weight loss either at the completion of the active intervention or at follow-up was shown for any of these intervention combinations.

### Limitations in the available literature

We identified several important limitations in the literature relative to addressing our primary question regarding the effect of the method of weight loss, i.e., energy restriction, exercise (aerobic or resistance), and the various combinations on long-term changes in weight, body composition or chronic disease risk factors in overweight or obese adults. The literature is limited by a lack of randomized trials that have addressed this question. For this review, we intended to evaluate trials with at least a 6 month active weight loss intervention followed by at least 12 months of active or passive weight maintenance. However, we were able to identify only 3 randomized trials that satisfied these criteria [77], [80], [89]. These trials were not statistically powered to address the question of interest and 2 of these trials reported on a final sample that represented ≤60% of participants randomized at baseline [77], [89]. Expanding our inclusion criteria to include randomized trials with active interventions and follow-up periods of any duration identified only 6 long-term trials (>6 mos.) and 14 trials with a follow-up period after weight loss. However, 43% of the weight loss/follow-up trials had follow-up periods of 6 months or less.

The available literature is also limited by the fact that the aerobic exercise that was prescribed, either alone, or in combination with a diet, was insufficient to produce significant weight loss. This is particularly problematic when evaluating the effect of the method of weight loss on weight regain, as greater weight loss following an active intervention is associated with greater weight loss at follow-up [12], [102]. Thus, the effect of exercise on long-term weight loss, or weight regain when exercise is prescribed during weight loss and/or during follow-up has not been adequately evaluated. Additional limitations include lack of data by sex or minority status, and lack of trials including resistance exercise. For example, of the 20 trials included in this review, 35% were conducted on all female samples, and 45% in samples that were predominantly female. Only 2 trials, one long-term (12-mos.) [82] and one follow-up (6 mos. weight loss, 6 mos. follow-up) [79] provided results separately by sex [79], [82]. Results of these trials suggest potential sex differences for change in weight and body composition which warrant additional investigation. Sixty percent of trials either failed to report minority representation or included no minorities, and only 3 trials evaluated the impact of resistance training either in combination with diet [84], [89] or in combination with diet and aerobic exercise [89], [93].

### Limitations of this review

Our conclusions should be cautiously interpreted as they are based on both data from a limited number of randomized trials, several with a high risk of one or more forms of bias. In addition, we did not contact authors to obtain missing data; however the author of one trial [78] was contacted for clarification regarding the statistical significance of one between group difference for data presented in a table.

## Conclusions

The present systematic review found limited evidence to suggest better long-term weight loss and more favorable long-term changes in body composition and chronic disease risk factors when diet is combined with aerobic exercise compared with either diet or aerobic exercise alone. However, no advantage for minimizing weight regain or changes in body composition or chronic disease risk factors following weight loss was shown for any weight loss mode. As previously discussed, the available literature on this topic is extremely limited and suffers numerous methodological shortcomings. Therefore, we recommend additional randomized trials to specifically evaluate the impact of mode of weight loss on changes in weight, body composition and chronic disease risk factors following both active weight loss and maintenance that include the following: 1) adequate statistical power to detect clinically significant differences in these outcomes both overall and by sex; 2) weight loss and maintenance phases of ≥6 months and ≥12 months, respectively; 3) supervised, verified exercise during weight loss of sufficient energy expenditure to achieve weight loss similar to that achieved in comparison groups (i.e. diet only or diet+exercise). 4) maintenance periods with and without prescribed exercise; and 5) resistance training.

## Supporting Information

Checklist S1 **PRISMA checklist.**
(TIFF)
