# Supplementary figures and images for: Does the Method of Weight Loss Effect Long-Term Changes in Weight, Body Composition or Chronic Disease Risk Factors in Overweight or Obese Adults? A Systematic Review
Source: PLoS One. 2014 Oct 15;9(10):e109849. doi: 10.1371/journal.pone.0109849 (PMC4198137; doi:10.1371/journal.pone.0109849)

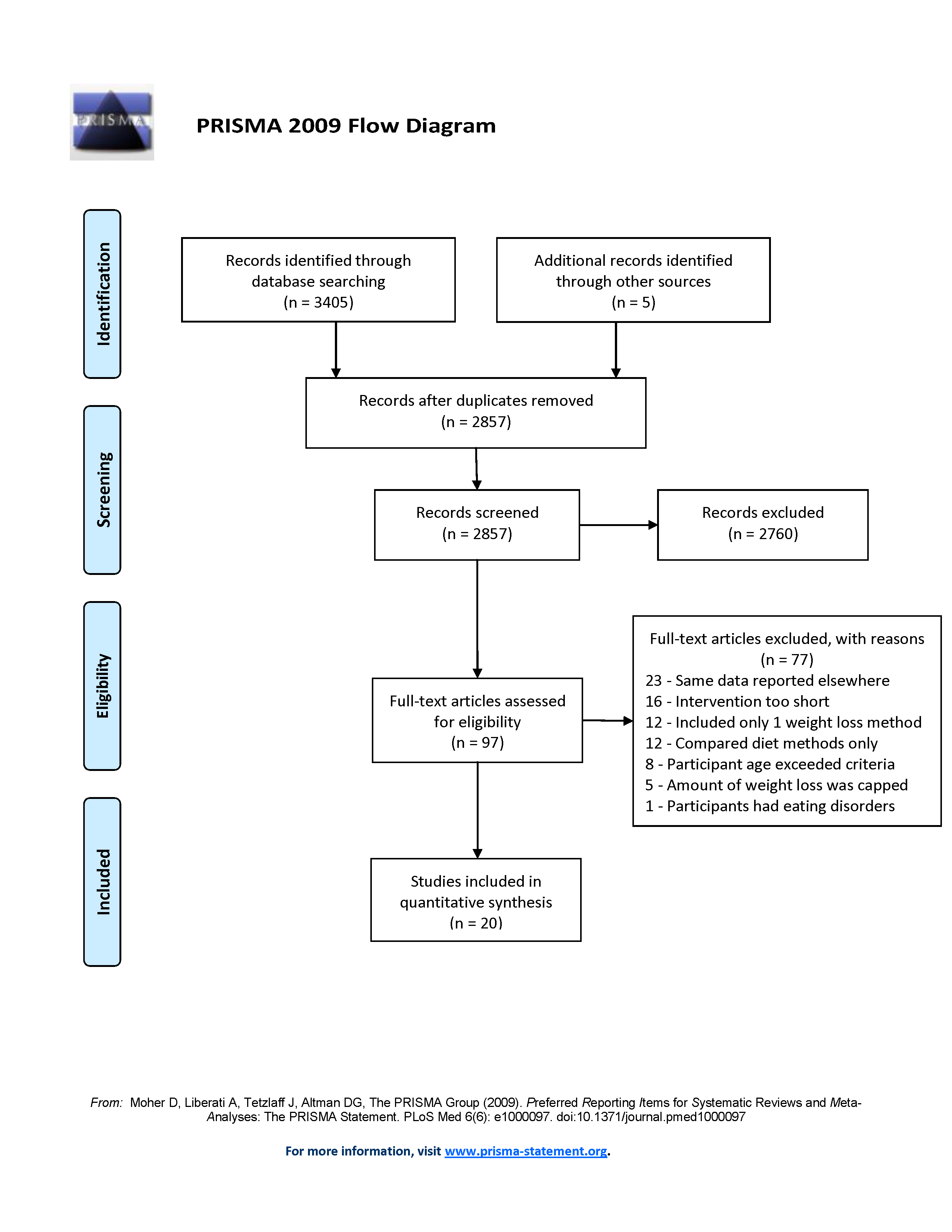

Supplement: Checklist S1 — PRISMA checklist. (TIFF) [file pone.0109849.s001.tiff]
